# A significant, functional and replicable risk *KTN1* variant block for schizophrenia

**DOI:** 10.1038/s41598-023-27448-z

**Published:** 2023-03-08

**Authors:** Qiao Mao, Xiandong Lin, Qin Yin, Ping Liu, Yong Zhang, Shihao Qu, Jianying Xu, Wenhong Cheng, Xinqun Luo, Longli Kang, Reyisha Taximaimaiti, Chengchou Zheng, Huihao Zhang, Xiaoping Wang, Honggang Ren, Yuping Cao, Jie Lin, Xingguang Luo

**Affiliations:** 1 Department of Psychosomatic Medicine, People’s Hospital of Deyang City, Deyang, 618000 Sichuan China; 2 Laboratory of Radiation Oncology and Radiobiology, Fujian Provincial Cancer Hospital, the Teaching Hospital of Fujian Medical University, Fuzhou, 350014 Fujian China; 3 Department of Respiratory and Critical Care Medicine, Hubei Provincial Hospital of Integrated Chinese and Western Medicine, Wuhan, 430000 Hubei China; 4 Tianjin Mental Health Center, Tianjin, 300222 China; 5 Zhuhai Center for Maternal and Child Health Care, Zhuhai, Guangdong 519001 China; 6 Shanghai Mental Health Center, Shanghai Jiao Tong University School of Medicine, Shanghai, 200030 China; 7 Department of Neurosurgery, The First Hospital, Fujian Medical University, Fuzhou, 350004 Fujian China; 8 Key Laboratory for Molecular Genetic Mechanisms and Intervention Research On High Altitude Diseases of Tibet Autonomous Region, Xizang Minzu University School of Medicine, Xiangyang, 712082 Shaanxi China; 9 Department of Neurology, Shanghai Tongren Hospital, Shanghai Jiao Tong University, Shanghai, 200080 China; 10 Minqing Psychiatric Hospital, Minqing, 350800 Fujian China; 11 The First Affiliated Hospital, Fujian Medical University, Fuzhou, 350001 China; 12 Department of Internal Medicine, Tongji Medical College, Huazhong University of Science and Technology, Wuhan, China; 13 Department of Psychiatry, Second Xiangya Hospital, Central South University, China National Clinical Research Center On Mental Disorders, China National Technology Institute On Mental Disorders, Changsha, 410011 Hunan China; 14 Fujian Center for Disease Control and Prevention, Fuzhou, 350012 Fujian China; 15 Fujian Institute of Preventive Medicine, Fuzhou, 350012 Fujian China; 16 Beijing Huilongguan Hospital, Peking University Huilongguan School of Clinical Medicine, Beijing, 100096 China; 17 Department of Neurology, The 1st People’s Hospital, Shanghai Jiaotong University School of Medicine, Shanghai, 201620 USA

**Keywords:** Genetic markers, Schizophrenia

## Abstract

Cortical and subcortical structural alteration has been extensively reported in schizophrenia, including the unusual expansion of gray matter volumes (GMVs) of basal ganglia (BG), especially putamen. Previous genome-wide association studies pinpointed kinectin 1 gene (*KTN1*) as the most significant gene regulating the GMV of putamen. In this study, the role of *KTN1* variants in risk and pathogenesis of schizophrenia was explored. A dense set of SNPs (n = 849) covering entire *KTN1* was analyzed in three independent European- or African-American samples (n = 6704) and one mixed European and Asian Psychiatric Genomics Consortium sample (n = 56,418 cases vs. 78,818 controls), to identify replicable SNP-schizophrenia associations. The regulatory effects of schizophrenia-associated variants on the *KTN1* mRNA expression in 16 cortical or subcortical regions in two European cohorts (n = 138 and 210, respectively), the total intracranial volume (ICV) in 46 European cohorts (n = 18,713), the GMVs of seven subcortical structures in 50 European cohorts (n = 38,258), and the surface areas (SA) and thickness (TH) of whole cortex and 34 cortical regions in 50 European cohorts (n = 33,992) and eight non-European cohorts (n = 2944) were carefully explored. We found that across entire *KTN1*, only 26 SNPs within the same block (r^2^ > 0.85) were associated with schizophrenia across ≥ 2 independent samples (7.5 × 10^–5^ ≤ p ≤ 0.048). The schizophrenia-risk alleles, which increased significantly risk for schizophrenia in Europeans (q < 0.05), were all minor alleles (*f* < 0.5), consistently increased (1) the *KTN1* mRNA expression in 12 brain regions significantly (5.9 × 10^–12^ ≤ p ≤ 0.050; q < 0.05), (2) the ICV significantly (6.1 × 10^–4^ ≤ p ≤ 0.008; q < 0.05), (3) the SA of whole (9.6 × 10^–3^ ≤ p ≤ 0.047) and two regional cortices potentially (2.5 × 10^–3^ ≤ p ≤ 0.042; q > 0.05), and (4) the TH of eight regional cortices potentially (0.006 ≤ p ≤ 0.050; q > 0.05), and consistently decreased (1) the BG GMVs significantly (1.8 × 10^–19^ ≤ p ≤ 0.050; q < 0.05), especially putamen GMV (1.8 × 10^–19^ ≤ p ≤ 1.0 × 10^–4^; q < 0.05, (2) the SA of four regional cortices potentially (0.010 ≤ p ≤ 0.048), and (3) the TH of four regional cortices potentially (0.015 ≤ p ≤ 0.049) in Europeans. We concluded that we identified a significant, functional, and robust risk variant block covering entire *KTN1* that might play a critical role in the risk and pathogenesis of schizophrenia.

## Introduction

Schizophrenia is a complex psychiatric disorder characterized by severe emotional, cognitive and social dysfunction. It usually has delusions, auditory verbal hallucinations, visual hallucinations, paranoia, emotional withdrawal, difficulty focusing, etc. Neuroimaging studies have reported widespread regional alteration throughout the brain in schizophrenia. Overall, the volumes or thickness of cortices are significantly reduced in schizophrenia, which has been consistently reported by most published studies, including frontal [prefrontal^1^, superior frontal Brodmann Areas BA9 and BA10^2–4^, middle frontal BA9^3,5^, rostral middle frontal^6^, and inferior frontal (inferior frontal^5^, orbital inferior frontal^7^, and orbito-frontal cortices^8^, and pars triangularis^9^ and inferior frontal sulci^10^)], temporal (inferior temporal^11–13^, middle temporal^7,11,12,14,15^, and superior temporal^3,11,12,16–22^), parietal (precuneus^23^), occipital (fusiform^7,11,13,24–26^ and lingual gyri^27,28^), and insular^29–33^ cortices, and limbic system (anterior^8,10^ and posterior cingulate gyri^8,10^, and amygdala^1,34–36^, hippocampus^1,7,34,36–38^ and thalamus^39^). Surprisingly, the gray matter volumes (GMVs) of basal ganglia (BG) (caudate nucleus^1,40–43^, putamen^40,44,45^, pallidum^40,46^, nucleus accumbens^47^, and substantia nigra^48,49^) have been reported to significantly increase in schizophrenia by most published studies, with only a couple of exception^34,39^. Among the five BG structures, enlargement of caudate nucleus and putamen in schizophrenia has been consistently reported to be the two most significant and important neural markers of schizophrenia^1,40–45,50–53^. The patients with enlarged putamen are usually sensitive to and benefit from treatment with antipsychotics that block dopamine neurotransmission^44,45,50^, as evidenced by the findings that patients with good treatment outcomes have larger putamen than those with poor outcomes or healthy controls, in support of enlarged putamen as a physiological correlate of neuroleptic responsiveness and a predictor of treatment outcome^44,45,50^.

The distinct alteration between cortices and subcortical BG in schizophrenia suggests that some schizophrenia-risk genes might regulate these structures. A genome-wide association study (GWAS) on GMVs of seven subcortical structures, including -BG (nucleus accumbens, caudate nucleus, putamen, and pallidum) and limbic system (amygdala, hippocampus and thalamus), identified that five genetic variants significantly influenced the volumes of putamen (*KTN1*, *DCC*, *BCL2L1* and *DLG2*) and caudate nucleus (*FAT3*)^54^. The strongest effect was found between putamen and rs945270 at 3′ flanking to *KTN1*. *KTN1* encodes kinectin 1 receptor, which regulates neuronal cell shape and volume^54–57^, and thus kinectin 1 expression in BG may regulate the volumes of these structures.

*KTN1* has been reported to play important roles in many neuropsychiatric or neurodegenerative diseases/phenotypes, including attention-deficit/hyperactivity disorder (ADHD)^58,59^, Parkinson’s disease (PD)^60–63^, heroin dependence^64^, marijuana dependence^65^, alcohol dependence^66^, and cognitive dysfunction in the elderly^67^. However, the role of *KTN1* in schizophrenia has not been explored yet. In this study, we examined a dense set of SNPs across entire *KTN1*, in order to identify variants that were associated with schizophrenia consistently across independent samples, which reduced false positives and improved the reliability of findings. And then, we explored the regulatory effects of these schizophrenia-risk variants on *KTN1* mRNA expression, GMVs of subcortical structures, and surface area (SA) and thickness (TH) of different cortical regions throughout whole brain.

## Materials and methods

### Subjects

Three independent samples, including two European and one African-American ones, were included for SNP-schizophrenia association analysis. Sample #1 included 1351 European-American patients with schizophrenia and 1378 healthy European-American controls. Sample #2 included a total of 1826 European parent–offspring trio subjects with 621 offspring with schizophrenia. Sample #3 included 1195 African-American patients with schizophrenia and 954 healthy African-American controls.

All subjects were at least 18 years old. Affected subjects met DSM-IV criteria for schizophrenia^68^. Patients with neurological disorders, substance use disorders, or mental retardation were excluded. Controls were free of schizophrenia, schizoaffective disorder, bipolar disorder, major depressive disorder, and psychotic symptoms including auditory hallucination and persecutory delusion. All subjects signed written informed consents before participating in the study. All study procedures were reviewed and approved by the Human Investigation Committee of Yale University (HIC#: 1007007175). Detailed demographic data for these samples have been published in previous studies^69–74^. All methods were carried out in accordance with relevant guidelines and regulations.

### SNP-disease association analysis

**Figure 1:**
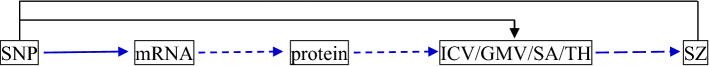
Regulatory pathway from SNP to schizophrenia. [Solid lines, pair-wise associations examined in the present study; dash lines, potential pair-wise associations predicted by literatures; arrows, regulatory effect directions; ICV, intracranial volume; GMV, subcortical gray matter volume; SA, cortical surface area; TH, cortical thickness; SZ, schizophrenia].

The design of this study is illustrated in Fig. 1, which showed the links between different methods/datasets. The statistical associations between SNPs and diseases were tested first, then the biological functions of the disease-risk SNPs were examined including their regulatory effects on mRNA expression in brains and brain structural measurements, which could validate that the SNP-disease associations were biological but not only statistical ones.

Samples #1 and #3 were genotyped on AFFYMETRIX AFFY_6.0 microarray platform, and Samples #2 was genotyped on Agilent SureSelect Human All Exon v.2 microarray platform. To make the genetic marker sets consistent across different samples, we imputed the untyped SNPs across the entire *KTN1* region using the same reference panels of 1000 Genome Project and HapMap3 Project data by the program IMPUTE2^75^. A total of 849 imputed SNPs covering entire open reading frame (ORF) of *KTN1*, 137 kb regulatory region at 5′ flanking *KTN1* that reaches the 3′-transcription termination site (TTS) of nearby *FBXO34* gene, and 433 kb regulatory region at 3′ flanking *KTN1* that reaches the 5′-transcription start site (TSS) of nearby *PELI2* gene, were analyzed.

Before statistical analysis, we stringently cleaned the phenotype and genotype data, as described in detail previously^74,76^. In brief, subjects with missing diagnosis, missing race, or a missing genotype call rate ≥ 2% across all SNPs were excluded. Furthermore, we excluded SNPs with an overall missing genotype call rate ≥ 2% across all subjects. The SNPs with minor allele frequencies ≤ 0.05 in affected subjects, or in Hardy–Weinberg disequilibrium (p < 0.001) in unaffected subjects were excluded.

The allele frequencies of SNPs were compared between individuals with schizophrenia and controls using Fisher exact test, or between “transmitted” and “untransmitted” using “–dfam” as implemented in PLINK^77^, to identify the statistical SNP-disease associations. Multiple comparisons in each Sample were corrected by false discovery rate (FDR). Q-values for these analyses that adjusted p-values based on an optimized FDR approach were computed using the R package QVALUE. A q-value < 0.05 indicated statistical significance, and a p-value < 0.05 but q-value > 0.05 indicated nominal significance. A SNP-disease association with p < 0.05 across at least two samples was taken as a potential replicable association. These associations were also verified in other independent cohorts of Psychiatric Genomics Consortium (PGC) data^78^.

### *cis*-acting expression quantitative trait locus (*cis-*eQTL) analysis

We examined the potential regulatory effects of schizophrenia-risk variants identified above on the *KTN1* mRNA expression in human postmortem brains in a UK European cohort (n = 138) (i.e., BRAINEAC dataset)^79^ and in a European-American cohort (n = 210) (i.e., GTEx dataset)^80^ using *cis-*eQTL analysis. These subjects were free of neurodegenerative and neuropsychiatric disorders. In the UK European cohort, a total of 10 brain regions were analyzed, including cerebellar, prefrontal, occipital, and temporal cortices, hippocampus, medulla, putamen, substantia nigra, thalamus, and intralobular white matter. In the European-American cohort, a total of 11 brain regions were analyzed, including BG (putamen, caudate nucleus, nucleus accumbens, and substantia nigra), limbic system [anterior cingulate gyrus (BA24), amygdala, hippocampus, and hypothalamus], prefrontal cortex (BA9), and cerebellum. Normalized mRNA expression levels were compared between different alleles of each variant using t-test. Multiple comparisons in each brain region were corrected by FDR.

### Regulatory effect of risk variants on the total intracranial volume (ICV) and the GMVs of subcortical structures

The ICV in 18,713 European subjects (17 CHAGE + 29 ENIGMA2 cohorts)^81^ and the GMVs of BG (caudate nucleus, putamen, pallidum, and nucleus accumbens) and limbic system (amygdale, hippocampus, and thalamus) in 38,258 European subjects (14 CHAGE + 35 ENIGMA2 + 1 UKBB cohorts)^54,82^ were measured by structural magnetic resonance imaging (MRI), following a standardized protocol procedure. GMVs were calculated using the brain segmentation software packages: FIRST^83^ or FreeSurfer^84^. All subjects were genotyped using microarray and imputed based on the 1000 Genome Project genotype panels. Genetic homogeneity was assessed in each subject using multi-dimensional scaling (MDS) analysis.

The potential regulatory effects of schizophrenia-risk variants identified above on ICV and GMVs were analyzed using multiple linear regression analysis, controlling for age, sex, 4 MDS components, ICV (for non-ICV phenotypes) and diagnosis (when applicable; most subjects were free of neurodegenerative and neuropsychiatric disorders). Multiple testing in each brain region was corrected by FDR.

### Regulatory effect of risk variants on cortical SA and average TH

A total of 36,936 subjects were analyzed, including 33,992 Europeans (23,909 from 49 ENIGMA cohorts and 10,083 from the UK Biobank) and 2,944 non-European participants (eight cohorts)^85^. Measures of cortical SA and TH of these subjects were derived from in vivo whole brain T1-weighted MRI scans using FreeSurfer^84^. SA and TH were quantified for each subject across whole cortex and within 34 distinct gyral-defined regions in each brain hemisphere according to the Desikan-Killiany atlas^86^. SA was measured at the grey-white matter boundary, and TH was measured as the average distance between white matter and pial surfaces. The SA and TH of each cortex were analyzed separately, instead of volume, based on the radial unit hypothesis^87^ that different developmental mechanisms promoted SA expansion and TH increases. The total SA and average TH of each hemisphere was computed separately.

The potential regulatory effects of schizophrenia-risk variants identified above on a total of 70 traits (total SA, average TH, and SA and TH of 34 cortical regions averaged across right and left hemispheres) were analyzed by exploring the associations between these variants and traits. These associations were analyzed by multiple linear regression analyses, adjusting for the effects of sex, linear and nonlinear age effects, interactions between age and sex, ancestry (the first four MDS components), diagnostic status (when the cohort followed a case–control design), MRI acquisition orientation, and dummy variables for scanner (when multiple scanners were used at the same site). When regional SA and TH were analyzed, the global measure (total SA or average TH) was added as an additional covariate, to test for genetic influences specific to each region. Multiple testing in each brain region was corrected by FDR.

## Results

### Replicable associations between risk variants and schizophrenia

**Figure 2:**
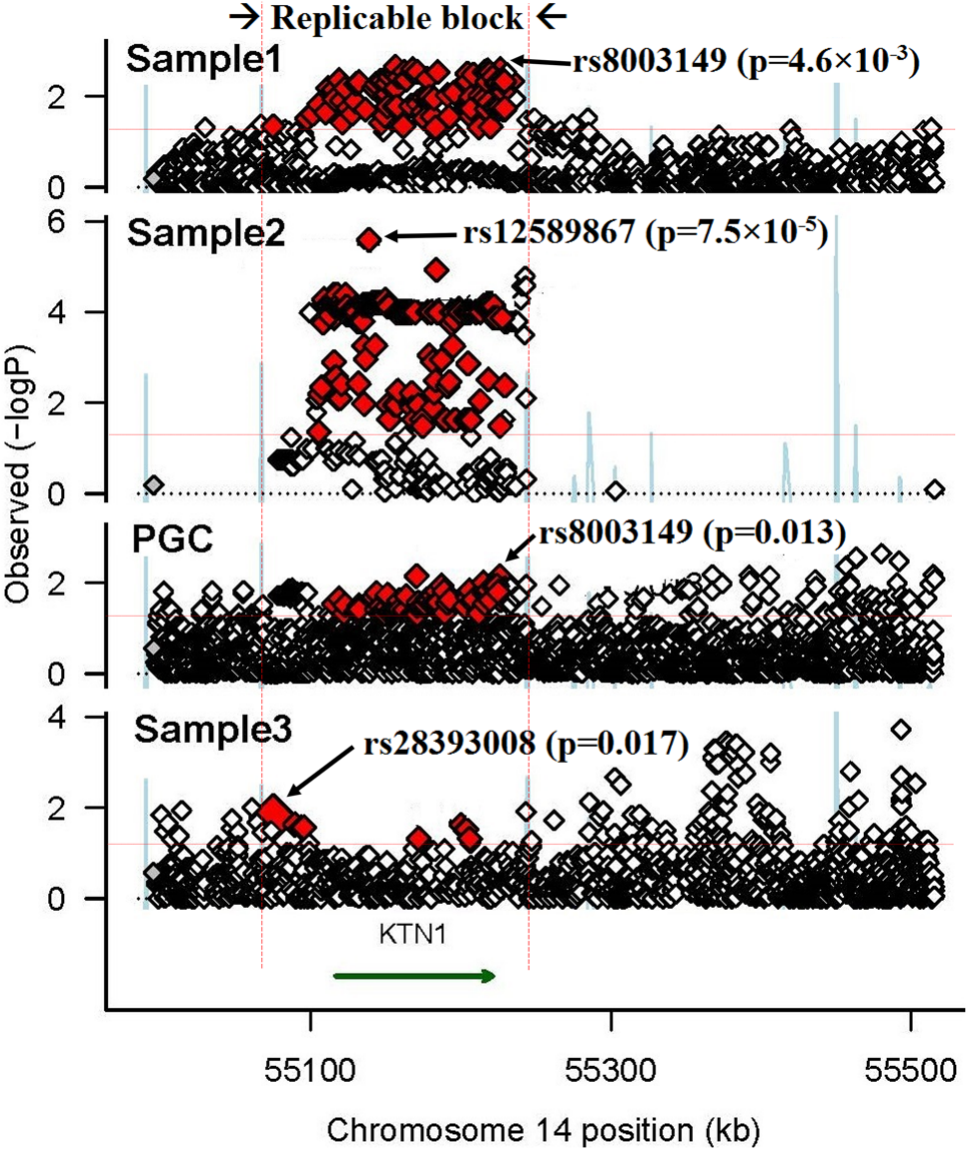
Regional association plots in four samples [X-axis, chromosome positions; Y-axis, −log(p) values; Sample names labeled at the left top corners correspond to Table 1; The most significant SNP with the lowest p-value in each plot is pointed by an arrow; Red diamonds are those SNPs with p < 0.05 and in high LD with one another; Red vertical dashed lines define the replicable association variant block; Red horizontal dashed line correspond to p = 0.05].

A total of 26 risk SNPs within the same variant block (r^2^ > 0.85), covering entire ORF of *KTN1* (Supplementary Fig. S1), were nominally associated with schizophrenia across at least two samples (7.5 × 10^–5^ ≤ p ≤ 0.049; Fig. 2); 25 of them were associated with schizophrenia across two independent European samples (7.5 × 10^–5^ ≤ p ≤ 0.049), which were significant after FDR correction in at least one sample (3.6 × 10^–5^ ≤ q ≤ 0.049). Five of these SNPs were also associated with schizophrenia in an African-American sample (0.017 ≤ p ≤ 0.048); and eight of them were also associated with schizophrenia in a mixed European and Asian sample (PGC data: 0.013 ≤ p ≤ 0.030; Table 1). The risk allele of each variant, which had significantly higher frequency in cases (or “transmitted” group) than controls (or “untransmitted” group), was the same one across different European samples, but the opposite one in African-Americans. All risk alleles in Europeans were minor alleles (*f* < 0.5), but major alleles in Africans (*f* > 0.5; Table 1).

**Table 1:** p/q [allelic frequency] values for associations between *KTN1* SNPs and schizophrenia in four samples.

SNP	Genomic Position	Risk allele	Sample #1	Sample #2	PGC	Risk allele	Sample #3
			European-American	European	European + Asian		African–American
			Cases: n = 1,351	Offspring: 621	Cases: 56,418		Cases: 1,195
			Controls: n = 1,378	Total: 1,826	Controls: 78,818		Controls: 954
rs28393008	55,074,996	T	0.046 [0.400/0.368]	–	> 0.05 [0.434/0.432]	C	0.017 [0.611/0.561]
rs8007832	55,105,332	C	0.015 [0.377/0.345]	0.044/**9.7 × 10**^**–3**^ [0.203/0.155]	> 0.05 [0.367/0.365]	–	> 0.05
rs12589867	55,115,552	T	7.2 × 10^–3^ [0.243/0.210]	7.5 × 10^–5^/**3.6 × 10**^**–5**^ [0.200/0.111]	0.030 [0.303/0.298]	–	> 0.05
rs10146870	55,115,685	C	0.012 [0.381/0.348]	2.8 × 10^–3^/**7.8 × 10**^**–4**^ [0.329/0.238]	> 0.05 [0.371/0.358]	–	> 0.05
rs4457912	55,120,649	A	0.012 [0.383/0.350]	3.9 × 10^–3^/**1.0 × 10**^**–3**^ [0.242/0.167]	> 0.05 [0.375/0.371]	–	> 0.05
rs8008764	55,151,228	G	0.017 [0.379/0.348]	0.011/**2.7 × 10**^**–3**^ [0.290/0.217]	> 0.05 [0.371/0.369]	–	> 0.05
rs7154548	55,155,943	C	0.017 [0.379/0.348]	0.011/**2.7 × 10**^**–3**^ [0.290/0.217]	> 0.05 [0.377/0.374]	–	> 0.05
rs12897227	55,157,544	A	0.017 [0.380/0.348]	0.011/**2.7 × 10**^**–3**^ [0.290/0.217]	> 0.05 [0.371/0.368]	–	> 0.05
rs28581272	55,166,881	A	0.016 [0.379/0.347]	0.011/**2.7 × 10**^**–3**^ [0.290/0.217]	> 0.05 [0.359/0.357]	–	> 0.05
rs34706911	55,171,772	T	0.015 [0.375/0.342]	0.011/**2.7 × 10**^**–3**^ [0.290/0.217]	> 0.05 [0.362/0.360]	A	0.048 [0.085/0.036]
rs7156566	55,174,504	T	0.026 [0.246/0.217]	0.033/**7.2 × 10**^**–3**^ [0.285/0.224]	0.030 [0.292/0.289]	–	> 0.05
rs12434799	55,178,927	G	0.010 [0.231/0.193]	9.0 × 10^–4^/**2.6 × 10**^**–4**^ [0.163/0.095]	0.024 [0.308/0.302]	–	> 0.05
rs2274078	55,183,203	T	0.049 [0.208/0.178]	3.2 × 10^–3^/**8.8 × 10**^**–4**^ [0.262/0.184]	0.027 [0.294/0.290]	–	> 0.05
rs12888809	55,191,180	T	9.5 × 10^–3^ [0.189/0.157]	4.3 × 10^–3^/**1.1 × 10**^**–3**^ [0.237/0.164]	0.028 [0.293/0.289]	–	> 0.05
rs35094494	55,192,442	A	0.028 [0.232/0.207]	3.5 × 10^–3^/**9.4 × 10**^**–4**^ [0.237/0.163]	0.023 [0.293/0.289]	–	> 0.05
rs3765516	55,195,951	A	8.3 × 10^–3^ [0.386/0.351]	0.024/**5.3 × 10**^**–3**^ [0.356/0.283]	> 0.05 [0.363/0.360]	–	> 0.05
rs12886475	55,202,936	T	0.019 [0.373/0.341]	0.024/**5.3 × 10**^**–3**^ [0.356/0.283]	> 0.05 [0.361/0.359]	G	0.030 [0.091/0.043]
rs7150285	55,203,695	T	0.016 [0.377/0.345]	0.024/**5.3 × 10**^**–3**^ [0.356/0.283]	> 0.05 [0.362/0.361]	C	0.030 [0.090/0.043]
rs12590936	55,204,802	T	4.7 × 10^–3^/**0.049** [0.193/0.160]	1.4 × 10^–3^/**3.8 × 10**^**–4**^ [0.069/0.029]	0.021 [0.292/0.289]	–	> 0.05
rs10444721	55,206,029	T	0.015 [0.378/0.346]	0.024/**5.3 × 10**^**–3**^ [0.356/0.283]	> 0.05 [0.361/0.359]	C	0.048 [0.091/0.047]
rs2296182	55,206,712	T	0.010 [0.378/0.344]	0.024/**5.3 × 10**^**–3**^ [0.356/0.283]	> 0.05 [0.357/0.356]	–	> 0.05
rs2296183	55,206,835	T	0.019 [0.379/0.348]	0.032/**0.012** [0.314/0.256]	> 0.05 [0.357/0.355]	–	> 0.05
rs12893162	55,212,885	A	9.9 × 10^–3^ [0.380/0.346]	9.1 × 10^–3^/**2.3 × 10**^**–3**^ [0.362/0.279]	> 0.05 [0.361/0.359]	–	> 0.05
rs17685650	55,218,433	A	0.014 [0.380/0.347]	3.1 × 10^–3^/**8.4 × 10**^**–4**^ [0.391/0.293]	> 0.05 [0.355/0.353]	–	> 0.05
rs8003149	55,226,257	C	4.6 × 10^–3^/**0.049** [0.283/0.249]	0.032/**7.1 × 10**^**–3**^ [0.064/0.037]	0.013 [0.301/0.297]	–	> 0.05
rs12895366	55,229,304	G	0.018 [0.387/0.356]	4.2 × 10^–3^/**1.1 × 10**^**–3**^ [0.296/0.214]	> 0.05 [0.364/0.363]	–	> 0.05

“– “, omitted alleles with p > 0.05. “–”, missing. The bold values indicate q < 0.05. The values in “[]” are allelic frequencies in cases/controls or transmitted/untransmitted. PGC, psychiatric genomics consortium. Risk alleles are minor alleles (*f* < 0.5) in Europeans but major alleles (*f* > 0.5) in Africans.

### The schizophrenia-risk variants significantly regulated the *KTN1* mRNA expression in brains

All of the 26 risk variants regulated the *KTN1* mRNA expression in several brain regions (5.9 × 10^–12^ ≤ p ≤ 0.050; Table 2), including suggestively in occipital cortex and putamen in BRAINEAC (6.2 × 10^–3^ ≤ p ≤ 0.047; q > 0.05), and suggestively (p < 0.05 but q > 0.05) or significantly (q < 0.05) in prefrontal cortex (BA9), anterior cingulate gyrus (BA24), cerebellum, amygdala, hippocampus, hypothalamus, and BG (caudate nucleus, nucleus accumbens, putamen and substantia nigra) in GTEx (5.9 × 10^–12^ ≤ p ≤ 0.050). The regulatory effects were potentially significant in putamen in both BRAINEAC and GTEx (4.5 × 10^–3^ ≤ p ≤ 0.050), however, in opposite directions; both were not significant after FDR correction though. Interestingly, all of the effective alleles that increased *KTN1* mRNA expression (t > 0) in GTEx were schizophrenia-risk alleles that increased risk for schizophrenia, and all effective alleles potentially increasing mRNA expression in BRAINEAC were schizophrenia-protective alleles. The effect direction for each SNP-mRNA association in BRAINEAC is illustrated in Fig. S2, and the effect size, i.e., normalized effect size (NES > 0), for each SNP-mRNA association in GTEx is presented in Fig. S3. Finally, no regulatory effects on *KTN1* mRNA expression in other (25%) brain regions were found, including medulla, temporal cortex, thalamus, and intralobular white matter (p > 0.05).

**Table 2:** p values for associations between schizophrenia-risk SNPs and *KTN1* mRNA expression in brain.

SNP		BRAINEAC (n = 138)		GTEx (n = 210)												
	Effective allele	Cortex	Basal ganglia	Effective allele	Cortex		Limbic system				Basal ganglia				Cerebellum	
		Occipital	Putamen		Cortex	Frontal	Anterior cingulate	Amygdala	Hippo-campus	Hypo-thalamus	Caudate	Putamen	Nucleus accumbens	Substantia nigra	Cerebellar Hemisphere	Cerebellum
rs28393008	C	6.2 × 10^–3^	–	T	**1.9 × 10**^**–3**^	4.7 × 10^–3^	4.7 × 10^–3^	4.7 × 10^–3^	4.7 × 10^–3^	–	**3.9 × 10**^**–4**^	0.05	**1.6 × 10**^**–3**^	0.05	**8.2 × 10**^**–4**^	**1.1 × 10**^**–5**^
rs8007832	T	0.013	0.026	C	**1.6 × 10**^**–3**^	**1.6 × 10**^**–3**^	**1.6 × 10**^**–3**^	**1.6 × 10**^**–3**^	**1.6 × 10**^**–3**^	–	**9.0 × 10**^**–5**^	0.02	**1.2 × 10**^**–4**^	–	**1.6 × 10**^**–3**^	**1.7 × 10**^**–6**^
rs12589867	G	0.019	–	T	**1.8 × 10**^**–4**^	2.5 × 10^–3^	2.5 × 10^–3^	2.5 × 10^–3^	2.5 × 10^–3^	5.8 × 10^–3^	**2.5 × 10**^**–3**^	5.8 × 10^–3^	**2.5 × 10**^**–5**^	5.8 × 10^–3^	**1.3 × 10**^**–4**^	**2.1 × 10**^**–11**^
rs10146870	G	0.013	0.023	C	**1.3 × 10**^**–3**^	**1.3 × 10**^**–3**^	**1.3 × 10**^**–3**^	**1.3 × 10**^**–3**^	**1.3 × 10**^**–3**^	–	**5.3 × 10**^**–5**^	0.02	**7.2 × 10**^**–5**^	–	**1.3 × 10**^**–3**^	**2.2 × 10**^**–6**^
rs4457912	G	0.015	0.022	A	**5.1 × 10**^**–4**^	**5.1 × 10**^**–4**^	**5.1 × 10**^**–4**^	**5.1 × 10**^**–4**^	**5.1 × 10**^**–4**^	–	**9.5 × 10**^**–5**^	0.02	**1.2 × 10**^**–4**^	–	**5.1 × 10**^**–4**^	**4.7 × 10**^**–6**^
rs8008764	T	0.047	0.025	G	**8.7 × 10**^**–4**^	**8.7 × 10**^**–4**^	**8.7 × 10**^**–4**^	**8.7 × 10**^**–4**^	**8.7 × 10**^**–4**^	–	**2.7 × 10**^**–4**^	0.03	**1.7 × 10**^**–4**^	–	**8.7 × 10**^**–4**^	**1.9 × 10**^**–5**^
rs7154548	T	0.047	0.026	C	**7.3 × 10**^**–4**^	**7.3 × 10**^**–4**^	**7.3 × 10**^**–4**^	**7.3 × 10**^**–4**^	**7.3 × 10**^**–4**^	–	**9.6 × 10**^**–5**^	0.02	**6.9 × 10**^**–5**^	–	**7.3 × 10**^**–4**^	**4.4 × 10**^**–6**^
rs12897227	G	0.046	0.026	A	**8.7 × 10**^**–4**^	**8.7 × 10**^**–4**^	**8.7 × 10**^**–4**^	**8.7 × 10**^**–4**^	**8.7 × 10**^**–4**^	–	**3.0 × 10**^**–4**^	0.02	**1.4 × 10**^**–4**^	–	**8.7 × 10**^**–4**^	**1.1 × 10**^**–5**^
rs28581272	T	0.046	0.026	A	**6.1 × 10**^**–4**^	**6.1 × 10**^**–4**^	**6.1 × 10**^**–4**^	**6.1 × 10**^**–4**^	**6.1 × 10**^**–4**^	–	**1.5 × 10**^**–4**^	0.05	**9.4 × 10**^**–5**^	–	**6.1 × 10**^**–4**^	**6.5 × 10**^**–6**^
rs34706911	A	0.039	0.029	T	**5.8 × 10**^**–4**^	**5.8 × 10**^**–4**^	**5.8 × 10**^**–4**^	**5.8 × 10**^**–4**^	**5.8 × 10**^**–4**^	–	**6.3 × 10**^**–5**^	0.03	**1.1 × 10**^**–4**^	–	**5.8 × 10**^**–4**^	**4.7 × 10**^**–6**^
rs7156566	G	*0.053*	–	T	**2.4 × 10**^**–5**^	**9.1 × 10**^**–4**^	**9.1 × 10**^**–4**^	**9.1 × 10**^**–4**^	**9.1 × 10**^**–4**^	0.011	**9.1 × 10**^**–4**^	0.011	**5.0 × 10**^**–5**^	0.011	**2.1 × 10**^**–5**^	**5.9 × 10**^**–12**^
rs12434799	A	*0.053*	–	G	**8.6 × 10**^**–4**^	**8.6 × 10**^**–4**^	**8.6 × 10**^**–4**^	**8.6 × 10**^**–4**^	**8.6 × 10**^**–4**^	0.023	**8.6 × 10**^**–4**^	0.023	**3.6 × 10**^**–5**^	0.023	**1.6 × 10**^**–4**^	**3.4 × 10**^**–11**^
rs2274078	A	*0.051*	–	T	**9.0 × 10**^**–4**^	**9.0 × 10**^**–4**^	**9.0 × 10**^**–4**^	**9.0 × 10**^**–4**^	**9.0 × 10**^**–4**^	0.034	**9.0 × 10**^**–4**^	0.034	**3.4 × 10**^**–5**^	0.034	**6.9 × 10**^**–5**^	**5.4 × 10**^**–11**^
rs12888809	C	*0.051*	–	T	**2.5 × 10**^**–5**^	**4.8 × 10**^**–4**^	**4.8 × 10**^**–4**^	**4.8 × 10**^**–4**^	**4.8 × 10**^**–4**^	4.5 × 10^–3^	**4.8 × 10**^**–4**^	4.5 × 10^–3^	**9.3 × 10**^**–5**^	4.5 × 10^–3^	**3.7 × 10**^**–5**^	**2.0 × 10**^**–11**^
rs35094494	G	*0.051*	–	A	**6.1 × 10**^**–5**^	0.014	0.014	0.014	**7.5 × 10**^**–4**^	4.5 × 10^–3^	**1.8 × 10**^**–3**^	4.5 × 10^–3^	**6.2 × 10**^**–5**^	4.5 × 10^–3^	**6.2 × 10**^**–5**^	**3.4 × 10**^**–11**^
rs3765516	C	0.043	0.028	A	**1.4 × 10**^**–3**^	–	–	–	**5.3 × 10**^**–4**^	–	**1.5 × 10**^**–4**^	0.04	**1.5 × 10**^**–4**^	–	0.01	**4.7 × 10**^**–6**^
rs12886475	G	0.030	0.039	T	**2.0 × 10**^**–3**^	–	–	–	**5.7 × 10**^**–4**^	–	**8.4 × 10**^**–5**^	0.04	**1.3 × 10**^**–4**^	–	0.02	**5.8 × 10**^**–6**^
rs7150285	C	0.040	0.030	T	**1.6 × 10**^**–3**^	–	–	–	**9.5 × 10**^**–4**^	–	**6.6 × 10**^**–5**^	0.02	**1.8 × 10**^**–4**^	–	0.02	**2.7 × 10**^**–5**^
rs12590936	C	*0.051*	–	T	**2.4 × 10**^**–5**^	0.020	0.020	0.020	**9.1 × 10**^**–4**^	0.011	**1.3 × 10**^**–3**^	0.011	**5.0 × 10**^**–5**^	0.011	**2.1 × 10**^**–5**^	**5.9 × 10**^**–12**^
rs10444721	C	0.045	0.027	T	2.1 × 10^–3^	–	–	–	**4.1 × 10**^**–4**^	–	**1.2 × 10**^**–4**^	0.04	**1.9 × 10**^**–4**^	–	0.01	**3.8 × 10**^**–6**^
rs2296182	C	0.045	0.027	T	0.010	–	–	–	**6.7 × 10**^**–4**^	–	**2.0 × 10**^**–4**^	0.03	**2.3 × 10**^**–4**^	–	0.02	**1.4 × 10**^**–5**^
rs2296183	G	0.045	0.027	T	0.010	–	–	–	**6.7 × 10**^**–4**^	–	**2.0 × 10**^**–4**^	0.03	**2.3 × 10**^**–4**^	–	0.02	**1.4 × 10**^**–5**^
rs12893162	G	0.045	0.027	A	**1.8 × 10**^**–3**^	–	–	0.050	**4.1 × 10**^**–4**^	–	**1.3 × 10**^**–4**^	0.03	**1.5 × 10**^**–4**^	–	0.02	**2.8 × 10**^**–6**^
rs17685650	C	0.045	0.027	A	4.3 × 10^–3^	–	0.040	0.040	**1.0 × 10**^**–3**^	–	**1.0 × 10**^**–4**^	0.02	**2.3 × 10**^**–4**^	–	0.01	**6.5 × 10**^**–6**^
rs8003149	T	0.042	–	C	**1.3 × 10**^**–5**^	0.014	0.014	0.014	**1.1 × 10**^**–3**^	0.049	**1.2 × 10**^**–3**^	0.04	**4.0 × 10**^**–5**^	0.049	**8.5 × 10**^**–5**^	**6.3 × 10**^**–11**^
rs12895366	T	0.038	0.037	G	**1.2 × 10**^**–3**^	–	–	–	**8.3 × 10**^**–4**^	–	**1.0 × 10**^**–4**^	0.03	**2.7 × 10**^**–4**^	–	0.01	**1.3 × 10**^**–5**^

“– “, p > 0.05. The bold p values correspond to q < 0.05. Some p values larger than but close to 0.05 are listed italic.

### The schizophrenia-risk variants significantly regulated the ICV and GMVs of subcortical structures

First of all, all of the 26 schizophrenia-risk variants regulated ICV in one sample (n = 18,713 Europeans; 6.1 × 10^–4^ ≤ p ≤ 0.008; Table 3); 16 of them were significant after FDR correction (q < 0.05). The effect size, i.e., Z-score (> 0), for each SNP-ICV association is presented in Fig. S4. Second, all or most of these variants significantly regulated the four BG GMVs (1.8 × 10^–19^ ≤ p ≤ 0.050; q < 0.05; Table 3), and many of these regulatory effects in each structure were replicable across two independent samples, including in putamen (1.8 × 10^–19^ ≤ p ≤ 1.0 × 10^–4^), caudate nucleus (3.9 × 10^–5^ ≤ p ≤ 0.033), pallidum (4.2 × 10^–7^ ≤ p ≤ 0.004), and nucleus accumbens (2.1 × 10^–4^ ≤ p ≤ 0.050). The regulatory effect on putamen was most significant among all structures. Consistently, all of the schizophrenia-risk alleles that increased risk for schizophrenia increased (z > 0) ICV, but decreased (z < 0) all GMVs of putamen, caudate nucleus, pallidum, and nucleus accumbens (Table 3). The effect size, i.e., Z-score or β (> 0), for each SNP-GMV association is presented in Fig. S5. Finally, no regulatory effects on limbic system (amygdale, hippocampus, and thalamus) were found (p > 0.05).

**Table 3:** p values for associations between schizophrenia-risk SNPs and ICV and GMVs of basal ganglia.

SNP	Effective allele	ICV	Effective allele	Putamen		Caudate		Pallidum		Nucleus accumbens	
		n = 18,713		29,451	13,688	29,451	13,688	29,451	13,688	29,451	13,688
rs28393008	T	5.3 × 10^–3^	C	**1.4 × 10**^**–13**^	**9.8 × 10**^**–9**^	**8.6 × 10**^**–4**^	5.1 × 10^–3^	**4.8 × 10**^**–5**^	**9.9 × 10**^**–5**^	**3.9 × 10**^**–4**^	3.1 × 10^–3^
rs8007832	C	**7.7 × 10**^**–4**^	T	**2.4 × 10**^**–17**^	**2.9 × 10**^**–10**^	**1.1 × 10**^**–4**^	4.5 × 10^–3^	**1.7 × 10**^**–6**^	**2.3 × 10**^**–6**^	**3.1 × 10**^**–4**^	0.021
rs12589867	T	5.7 × 10^–3^	G	**2.8 × 10**^**–7**^	**7.0 × 10**^**–5**^	0.025	–	4.0 × 10^–3^	**1.5 × 10**^**–5**^	–	–
rs10146870	C	**1.7 × 10**^**–3**^	G	**5.3 × 10**^**–18**^	–	**9.4 × 10**^**–5**^	–	**2.3 × 10**^**–6**^	–	**3.2 × 10**^**–4**^	–
rs4457912	A	3.1 × 10^–3^	G	**1.7 × 10**^**–18**^	**2.5 × 10**^**–10**^	**4.5 × 10**^**–5**^	5.2 × 10^–3^	**7.0 × 10**^**–7**^	**6.8 × 10**^**–6**^	**2.9 × 10**^**–4**^	0.020
rs8008764	G	**1.1 × 10**^**–3**^	T	**3.4 × 10**^**–17**^	**9.5 × 10**^**–10**^	**1.9 × 10**^**–4**^	0.016	**2.4 × 10**^**–6**^	**6.6 × 10**^**–6**^	**1.5 × 10**^**–3**^	–
rs7154548	C	**1.7 × 10**^**–3**^	T	**4.8 × 10**^**–18**^	**7.7 × 10**^**–10**^	**1.2 × 10**^**–4**^	0.016	**1.2 × 10**^**–6**^	**1.3 × 10**^**–5**^	**1.2 × 10**^**–3**^	–
rs12897227	A	**1.2 × 10**^**–3**^	G	**2.7 × 10**^**–17**^	**7.4 × 10**^**–10**^	**1.8 × 10**^**–4**^	0.017	**2.5 × 10**^**–6**^	**6.2 × 10**^**–6**^	**1.4 × 10**^**–3**^	–
rs28581272	A	**9.8 × 10**^**–4**^	T	**2.2 × 10**^**–18**^	–	**1.0 × 10**^**–4**^	–	**6.3 × 10**^**–7**^	–	**7.5 × 10**^**–4**^	–
rs34706911	T	**1.6 × 10**^**–3**^	A	**1.3 × 10**^**–18**^	–	**9.6 × 10**^**–5**^	–	**4.5 × 10**^**–7**^	–	**5.4 × 10**^**–4**^	–
rs7156566	T	5.5 × 10^–3^	G	**1.6 × 10**^**–7**^	**7.8 × 10**^**–5**^	0.029	–	**1.9 × 10**^**–3**^	**5.8 × 10**^**–6**^	–	–
rs12434799	G	6.5 × 10^–3^	A	**2.2 × 10**^**–7**^	**8.0 × 10**^**–5**^	0.022	–	**1.6 × 10**^**–3**^	**6.5 × 10**^**–6**^	–	–
rs2274078	T	7.5 × 10^–3^	A	**2.4 × 10**^**–7**^	–	0.024	–	2.1 × 10^–3^	–	–	–
rs12888809	T	5.8 × 10^–3^	C	**1.9 × 10**^**–7**^	**8.6 × 10**^**–5**^	0.030	–	2.4 × 10^–3^	**1.0 × 10**^**–5**^	–	–
rs35094494	A	5.0 × 10^–3^	G	**1.7 × 10**^**–7**^	**8.5 × 10**^**–5**^	0.029	–	2.3 × 10^–3^	**1.1 × 10**^**–5**^	–	–
rs3765516	A	**1.3 × 10**^**–3**^	C	**1.8 × 10**^**–18**^	**4.5 × 10**^**–10**^	**7.0 × 10**^**–5**^	0.012	**5.3 × 10**^**–7**^	**7.2 × 10**^**–6**^	**4.1 × 10**^**–4**^	0.045
rs12886475	T	**1.1 × 10**^**–3**^	G	–	**5.4 × 10**^**–10**^	–	0.017	**3.5 × 10**^**–6**^	**3.5 × 10**^**–6**^	6.1 × 10^–3^	–
rs7150285	T	**1.3 × 10**^**–3**^	C	**1.2 × 10**^**–18**^	**4.3 × 10**^**–10**^	**7.5 × 10**^**–5**^	0.011	**4.2 × 10**^**–7**^	**5.9 × 10**^**–6**^	**2.9 × 10**^**–4**^	0.039
rs12590936	T	4.2 × 10^–3^	C	**2.0 × 10**^**–7**^	**9.6 × 10**^**–5**^	0.033	–	3.1 × 10^–3^	**1.1 × 10**^**–5**^	–	–
rs10444721	T	**1.1 × 10**^**–3**^	C	**2.6 × 10**^**–17**^	**4.3 × 10**^**–9**^	**2.2 × 10**^**–4**^	0.032	**9.0 × 10**^**–7**^	**1.0 × 10**^**–5**^	**3.4 × 10**^**–4**^	–
rs2296182	T	**9.2 × 10**^**–4**^	C	**1.1 × 10**^**–17**^	**4.8 × 10**^**–10**^	**1.7 × 10**^**–4**^	0.018	**1.6 × 10**^**–6**^	**5.0 × 10**^**–6**^	**5.6 × 10**^**–4**^	–
rs2296183	T	**7.8 × 10**^**–4**^	G	**1.4 × 10**^**–17**^	**4.4 × 10**^**–10**^	**1.7 × 10**^**–4**^	0.016	**1.4 × 10**^**–6**^	**5.0 × 10**^**–6**^	**5.6 × 10**^**–4**^	–
rs12893162	A	**1.1 × 10**^**–3**^	G	**1.9 × 10**^**–18**^	**4.5 × 10**^**–10**^	**7.2 × 10**^**–5**^	0.012	**6.2 × 10**^**–7**^	**8.4 × 10**^**–6**^	**3.4 × 10**^**–4**^	0.045
rs17685650	A	**6.1 × 10**^**–4**^	C	**3.8 × 10**^**–18**^	**4.6 × 10**^**–10**^	**1.7 × 10**^**–4**^	0.014	**2.1 × 10**^**–6**^	**6.1 × 10**^**–6**^	**6.2 × 10**^**–4**^	–
rs8003149	C	0.011	T	**2.4 × 10**^**–8**^	**1.0 × 10**^**–4**^	0.012	–	**1.7 × 10**^**–3**^	**4.6 × 10**^**–5**^	–	–
rs12895366	G	**1.6 × 10**^**–3**^	T	**1.8 × 10**^**–19**^	**2.0 × 10**^**–10**^	**3.9 × 10**^**–5**^	8.6 × 10^–3^	**5.6 × 10**^**–7**^	**7.8 × 10**^**–6**^	2.1 × 10^–4^	0.050

“– “, p > 0.05. The bold p values correspond to q < 0.05. ICV, total intracranial volume; GMV, gray matter volume.

### The schizophrenia-risk variants regulated cortical SA

All or most of the schizophrenia-risk variants (excluding the missing rs7150285) potentially regulated the SA of whole brain, lingual gyrus, middle temporal cortex, precuneus, insula, and frontal pole (2.5 × 10^–3^ ≤ p ≤ 0.048; Table 4), although not significantly after FDR correction (q > 0.05). Overall, most of the schizophrenia-risk alleles that increased risk for schizophrenia potentially increased (β > 0) the SA of whole cortex (9.6 × 10^–3^ ≤ p ≤ 0.047). Regionally, all or most of these schizophrenia-risk alleles consistently potentially increased (β > 0) the SA of lingual gyrus and middle temporal cortex (2.5 × 10^–3^ ≤ p ≤ 0.042), but potentially decreased (β < 0) the cortical SA of precuneus, insula and frontal pole (0.010 ≤ p ≤ 0.048). Additionally, rs28393008 potentially decreased (β < 0) the SA of superior temporal cortex (p = 0.037; data not shown). Among these cortices, the precuneus, lingual gyrus, middle and superior temporal cortices are vicinages spatially. The effect size, i.e., β (> 0), for each SNP-SA association is presented in Fig. S6. Finally, no regulatory effects on other 28 regional cortices were found (p > 0.05).

**Table 4:** p values for associations between schizophrenia-risk SNPs and cortical surface areas and thickness.

SNP	Surface area (SA)								Thickness (TH)										
	Effect allele	Total	EU	EU		Total	Total	Total	Effect allele	EU	UKBB	EU	EU	UKBB	EU	UKBB	UKBB	Effect allele	UKBB
		n = 33,040	31,857	30,815		34,866	34,559	35,079		32,426	9,906	31,883	31,937	9,880	32,289	9,887	9,895		9,254
				Middle	Effect			Frontal		Frontal	Frontal	Superior	Rostral middle	Lateral	Pars	Isthmus	Inferior		
		Whole	Lingual	temporal	allele	Precuneus	Insula	pole		pole	pole	frontal	frontal	orbito–frontal	opercularis	cingulate	temporal		Pericalcarine
rs28393008	T	0.047	0.027	0.037	C	0.031	–	–	T	6.6 × 10^–3^	0.028	–	0.046	0.034	–	–	0.026	–	–
rs8007832	C	9.6 × 10^–3^	0.020	0.020	T	0.023	0.048	–	C	9.1 × 10^–3^	*0.053*	0.051	0.034	–	–	–	–	T	0.021
rs12589867	T	–	2.8 × 10^–3^	–	G	0.038	–	–	T	0.042	0.031	–	–	–	–	0.017	0.032	–	–
rs10146870	C	9.8 × 10^–3^	0.016	0.019	G	0.017	0.037	–	C	9.0 × 10^–3^	0.045	0.037	0.045	–	–	–	–	G	0.018
rs4457912	A	0.014	9.5 × 10^–3^	0.025	G	0.019	0.017	–	A	8.5 × 10^–3^	*0.058*	0.023	0.032	0.042	0.038	–	–	G	0.015
rs8008764	G	9.6 × 10^–3^	0.011	0.030	T	0.014	0.038	–	G	8.8 × 10^–3^	0.047	–	0.032	–	0.044	–	–	T	0.024
rs7154548	C	0.014	8.6 × 10^–3^	0.032	T	0.021	0.015	–	C	7.8 × 10^–3^	*0.057*	0.031	0.023	0.040	0.026	–	–	T	0.017
rs12897227	A	0.011	0.010	0.026	G	0.016	0.038	–	A	9.4 × 10^–3^	*0.052*	–	0.024	–	0.044	–	–	G	0.025
rs28581272	A	0.017	8.3 × 10^–3^	0.031	T	0.026	0.016	–	A	0.010	*0.062*	0.033	0.021	0.038	0.024	–	–	T	0.019
rs34706911	T	0.018	6.9 × 10^–3^	0.038	A	0.017	0.021	–	T	7.0 × 10^–3^	*0.069*	0.036	0.024	0.034	0.035	–	–	A	0.015
rs7156566	T	–	3.1 × 10^–3^	–	G	0.019	–	0.039	T	0.032	0.039	–	–	–	–	0.015	0.033	–	–
rs12434799	G	–	2.8 × 10^–3^	–	A	0.019	–	–	G	0.029	0.033	–	–	–	–	0.016	0.023	–	–
rs2274078	T	–	2.6 × 10^–3^	–	A	0.015	–	–	T	0.030	0.035	–	–	–	–	0.017	0.026	–	–
rs12888809	T	–	3.3 × 10^–3^	–	C	0.019	–	0.050	T	0.033	0.041	–	–	–	–	0.013	0.038	–	–
rs35094494	A	–	3.4 × 10^–3^	–	G	0.021	–	0.048	A	0.033	0.041	–	–	–	–	0.012	0.038	–	–
rs3765516	A	0.017	7.1 × 10^–3^	0.040	C	0.017	0.020	–	A	6.8 × 10^–3^	*0.068*	0.040	0.017	0.036	0.033	–	–	C	0.016
rs12886475	T	0.019	0.012	–	G	0.033	0.022	–	T	8.0 × 10^–3^	*0.072*	–	0.021	0.039	–	–	–	G	0.020
rs7150285	–	–	–	–	–	–	–	–	–	–	–	–	–	–	–	–	–	–	–
rs12590936	T	–	3.0 × 10^–3^	–	C	0.019	–	0.036	T	0.029	0.040	–	–	–	–	0.014	0.040	–	–
rs10444721	T	0.016	8.6 × 10^–3^	0.038	C	0.015	0.022	–	T	6.8 × 10^–3^	*0.066*	–	0.022	0.037	0.034	–	–	C	0.019
rs2296182	T	0.012	0.010	0.034	C	0.011	–	–	T	6.6 × 10^–3^	*0.054*	–	0.024	0.051	0.043	–	–	C	0.021
rs2296183	T	0.013	0.011	0.035	G	0.010	–	–	T	6.8 × 10^–3^	*0.056*	–	0.024	0.049	0.044	–	–	G	0.019
rs12893162	A	0.015	9.0 × 10^–3^	0.037	G	0.016	0.019	–	A	7.0 × 10^–3^	*0.069*	–	0.022	0.036	0.035	–	–	G	0.017
rs17685650	A	0.015	0.015	0.042	C	0.016	0.048	0.042	A	7.4 × 10^–3^	*0.062*	–	0.025	–	–	–	–	C	0.020
rs8003149	C	–	2.5 × 10^–3^	–	T	0.025	–	–	C	0.022	0.030	–	–	0.044	–	0.018	–	–	–
rs12895366	G	0.016	9.7 × 10^–3^	0.019	T	0.013	0.013	–	G	6.1 × 10^–3^	0.050	–	0.017	0.032	0.035	–	–	T	0.015

“– “, p > 0.05 or mising. Some p values larger than but close to 0.05 are listed italic. EU, Europeans; UKBB, UK biobank samples.

### The schizophrenia-risk variants regulated the average TH of cortices

All or most of the schizophrenia-risk variants (excluding the missing rs7150285) potentially regulated the TH of cortices spatially surrounding the frontal pole (frontal pole, superior frontal, rostral middle frontal, lateral orbito-frontal, and pars opercularis cortices) or the isthmus cingulate gyrus (isthmus cingulate, precuneus, and fusiform gyri, and pericalcarine, transverse temporal, inferior temporal, and paracentral cortices) (0.006 ≤ p ≤ 0.050; Table 4), although not significantly after FDR correction (q > 0.05).

Specifically, these schizophrenia-risk alleles potentially increased (β > 0) the TH of frontal pole cortex (0.006 ≤ p ≤ 0.050; Table 4), which was replicable across two independent samples. They also potentially increased (β > 0) the TH of superior frontal (0.023 ≤ p ≤ 0.050), rostral middle frontal (0.017 ≤ p ≤ 0.046), lateral orbito-frontal (0.032 ≤ p ≤ 0.050), and inferior temporal cortices (0.023 ≤ p ≤ 0.040), pars opercularis (0.024 ≤ p ≤ 0.044), and isthmus cingulate gyrus (0.012 ≤ p ≤ 0.018),. In addition, rs28393008 also potentially increased (β > 0) the TH of fusiform gyrus (p = 0.019; data not shown) that was next to the inferior temporal cortex. The effect directions of these variants were consistent across these regions. On the other hand, these schizophrenia-risk alleles potentially decreased (β < 0) the TH of pericalcarine cortex (0.015 ≤ p ≤ 0.025) that was next to lingual gyrus. In addition, rs28393008 also potentially decreased (β < 0) the TH of transverse temporal cortex, which was replicable across two independent samples (p = 0.023 and 0.049, respectively), precuneus (p = 0.042) and paracentral cortex (p = 0.016) (data not shown). The precuneus, lingual gyrus, and pericalcarine and transverse temporal cortices are all conjunctive with or near the isthmus cingulate gyrus and insula spatially. The effect size, i.e., β (> 0), for each SNP-TH association is presented in Fig. S7. Finally, no regulatory effects on other 22 regional cortices were found (p > 0.05).

## Summary

The minor alleles (*f* < 0.5) of all variants within the same block increased risk for schizophrenia in Europeans, but decreased risk in Africans. All or most of the schizophrenia-risk alleles suggestively or significantly increased (1) the *KTN1* mRNA expression in the prefrontal cortex (BA9), anterior cingulate gyrus (BA24), cerebellum, amygdala, hippocampus, hypothalamus, and BG (caudate nucleus, nucleus accumbens, putamen and substantia nigra) in GTEx, (2) the ICV, (3) the SA of whole brain cortex, (4) the SA of lingual gyrus and middle temporal cortex, and (5) the TH of frontal pole, superior frontal, rostral middle frontal, lateral orbito-frontal, and inferior temporal cortices, pars opercularis, and isthmus cingulate gyrus. In addition, rs28393008 within this block also increased the TH of fusiform gyrus. On the other hand, these alleles decreased (1) the *KTN1* mRNA expression in the occipital cortex and putamen in BRAINEAC, (2) all GMVs of putamen, caudate nucleus, pallidum, and nucleus accumbens, (3) the SA of precuneus, insula, and frontal pole and superior temporal cortices, and (4) the TH of precuneus, and pericalcarine, transverse temporal and paracentral cortices. After FDR correction, associations between SNPs and risk for schizophrenia in Europeans, *KTN1* mRNA expression in brain in GTEx, and ICV and BG GMVs remained significant. Finally, the mRNA expression in 25% of brain regions examined, and the GMVs of limbic system (amygdale, hippocampus, and thalamus) and other 18 regional cortices (~ 50%) were not significantly affected at all.

## Discussion

We identified 26 risk *KTN1* variants that were located within the same block, spanned entire *KTN1*, and were significantly associated with schizophrenia across at least two independent samples. These schizophrenia-risk variants had potential or significant regulatory effects on the *KTN1* mRNA expression in cortical or subcortical structures, the GMVs of subcortical structures, the SA and/or TH of about 50% cortices. The patterns of allele-schizophrenia associations and regulatory effects were highly consistent, replicable and robust, supporting that the *KTN1* variants may be biologically functional and play a critical role in the development of schizophrenia. These findings were majorly identified in Europeans in this study, and genetic difference between Europeans and Africans were also identified.

Globally, the schizophrenia-risk alleles (minor alleles) increased ICV and the SA of whole cortex. Regionally, these schizophrenia-risk *KTN1* alleles regulated the mRNA expression, GMVs, SA and/or TH widely across numerous brain regions, including both cortices and subcortical structures. The affected cortices spread over frontal lobe [frontal pole, superior frontal, middle frontal, inferior frontal (pars opercularis), paracentral, and lateral orbitofrontal cortices], temporal lobe (transverse temporal, superior temporal, middle temporal, and inferior temporal cortices), parietal lobe (precuneus), occipital lobe (occipital and pericalcarine cortices, and lingual and fusiform gyri), limbic system (anterior cingulate and isthmus of cingulate gyri), insula and cerebellum. The affected subcortical structures majorly included BG [striatum (putamen, caudate nucleus and nucleus accumbens), pallidum and substantia nigra] and limbic system (pallidum, nucleus accumbens, amygdala, hippocampus, and hypothalamus).

The most significant, consistent and reliable region regulated by the schizophrenia-risk alleles was frontal lobe, majorly prefrontal cortex, centering around the frontal pole. The schizophrenia-risk alleles increased the *KTN1* mRNA expression in prefrontal cortex (BA9) and the TH of frontal pole, superior frontal, rostral middle frontal, and lateral orbito-frontal cortices, and pars opercularis. Interestingly, the schizophrenia-risk alleles potentially decreased the SA of frontal pole, which was opposite to the effects on the TH, supporting the radial unit hypothesis that SA and TH have distinct neurodevelopmental origins. The frontal lobe contains most of dopamine neurons in cerebral cortices and the dopaminergic pathways have long been associated with the development of schizophrenia. The frontal cortex is usually involved in attention^88^, memory^89^, planning^90^, motivation^91^, cognitive process of decision-making^92^, emotion^93^, processing of speech and language, and self-awareness^94^. The prefrontal cortex is the largest component of frontal cortex, and is responsible for internal, purposeful mental action, reasoning or prefrontal synthesis, being involved in the higher-order mental alteration in schizophrenia.

The second most significant, highly consistent and reliable region regulated by the schizophrenia-risk alleles was BG. The schizophrenia-risk alleles suggestively or significantly increased the *KTN1* mRNA expression in BG (putamen, caudate nucleus, nucleus accumbens, and/or substantia nigra) in GTEx, but decreased the expression in BG (putamen) suggestively in BRAINEAC, and all four BG GMVs significantly. Among these four structures, the striatum (caudate nucleus, putamen and nucleus accumbens) was most affected, and the regulatory effects on the GMV of putamen were much more significant and robust than any other, consistent with the report that *KTN1* was the most significant gene regulating the GMV of putamen.

BG, critical components of the “cortico-BG-thalamo-cortical” loop, play an important role in cognition^95^ and emotion. Striatum receives excitatory glutamatergic input from cortices, and then sends output to other components of BG including pallidum and substantia nigra pars reticulate (SNr). The external globus pallidus (GPe) receives input from striatum, and sends inhibitory GABAergic output to subthalamic nucleus (STN); the internal globus pallidus (GPi) receives input from striatum, and sends inhibitory GABAergic output to thalamus. STN receives input from GPe and projects excitatory glutamatergic output to GPi or SNr. SNr sends inhibitory GABAergic output to thalamus. Finally, thalamus relays these outputs to cortices, completing the projection loops. The loops with projections to BG from prefrontal, lateral orbitofrontal, and temporal cortices, and anterior cingulate gyrus are called cognitive/associative pathways, playing a role in cognitive processing and frontal lobe functioning. The dysfunction of BG in these loops may result in uncontrolled behaviors as well as cognitive deficits of schizophrenia, similar to those that result from damage to prefrontal cortex^96^. In addition, the loops with projections to the limbic part of BG (nucleus accumbens and ventral pallidum) from anterior cingulate gyrus, hippocampus, and insula are called limbic circuit^97^, playing a central role in reward learning as well as cognition and frontal lobe functioning too, via the mesolimbic pathway that uses the neurotransmitter dopamine, and the mesocortical pathway. Overactive dopaminergic projection of mesolimbic pathway has been implicated in schizophrenia^98^. Our findings that schizophrenia-risk alleles regulated *KTN1* mRNA expression, and SA and TH of cortices in limbic system (amygdala, hippocampus, hypothalamus, and cingulate gyrus) and insula supported this implication.

Other three lobes, including the temporal, parietal, and occipital lobes, were also regulated by the schizophrenia-risk alleles. First, we found that the schizophrenia-risk alleles increased the SA of middle temporal cortex and the TH of inferior temporal cortex, but decreased the SA of superior temporal cortex and the TH of transverse temporal cortex. The alteration of these temporal regions may cause psychotic symptoms of schizophrenia in language processing, social cognition and auditory verbal hallucinations^99,100^. Second, we found that the schizophrenia-risk alleles decreased the SA and TH of precuneus. This parietal region has been involved in mental imagery concerning the self^101^, episodic memory^102^, and visuospatial imagery^103^. Finally, we found that the schizophrenia-risk alleles increased the SA of lingual gyrus and the TH of fusiform gyrus, but decreased the *KTN1* mRNA expression in occipital cortex (in BRAINEAC), and the TH of pericalcarine cortex. These occipital cortices are where the primary visual cortex is normally located, and responsible for visual and sensory information processing^104^. The occipital lesions can cause visual hallucinations of schizophrenia.

We found that the schizophrenia-risk alleles decreased the volumes of some cortices, including frontal [frontal pole (SA) and paracentral], temporal (superior and transverse temporal), parietal (precuneus), occipital (pericalcarine), and insular cortices, which was consistent with the previous findings that the volumes of these cortices in schizophrenia were reduced, suggesting that *KTN1* variants might play a dominant role in regulating the volumes in these areas. Conversely, we found that these schizophrenia-risk alleles increased the volumes of other cortices, including ICV, whole cortex, frontal [frontal pole (TH), superior frontal, rostral middle frontal, lateral orbito-frontal, and pars opercularis], temporal (middle and inferior temporal), and occipital (lingual and fusiform gyri) cortices, and limbic system (isthmus cingulate gyrus), which contradicted the previous findings that the volumes of these cortices were also reduced in schizophrenia. A hypothesis of compensation may be able to interpret these contradictory findings: First, other schizophrenia-risk non-*KTN1* genes might be dominant in these areas, significantly decreasing the volumes of cortices and thus the neurotransmission in the “cortico-BG-thalamo-cortical” loop. Next, as a compensatory response to reduction of excitatory glutamatergic output from cortices to BG, the expression of cortical volume-expanding proteins may be activated, in order to maintain neural transmission within this loop. One of these proteins is kinectin 1, as would be evidenced by our finding that the schizophrenia-risk alleles increased the *KTN1* mRNA expression in most cortices examined, which may then increase the kinectin expression in neurons, expand the cell sizes, and increase the volumes of the cortices. Finally, this expression activation of volume-expanding *KTN1* in cortices did not appear to restore the reduced volumes of cortices and neurotransmission of the loop resulting from other dominant schizophrenia-risk genes, suggesting that *KTN1* played a “recessive” role in these areas. This hypothesis well interpreted how the associations between schizophrenia-risk *KTN1* alleles and increased cortical volumes co-existed with the associations between schizophrenia and reduced cortical volumes in some brain areas.

Unexpectedly, the schizophrenia-risk alleles decreased the BG GMVs, contradictory to the previous findings of BG GMV enlargement in schizophrenia. This was highly possibly because other, but not the current, set of variants in the regulatory region 3′ flanking to *KTN1*, including rs945270^54^, dominantly increased the BG GMVs (Luo et al., unpublished data). As a compensatory response to the expansion of BG GMVs, the expression of some volume-shrinking *KTN1* alleles might be activated to shrink the BG GMVs, although this compensatory shrinkage did not restore the expansion of BG GMVs, suggesting a “recessive” role of these volume-shrinking *KTN1* alleles in BG GMVs. The current set of schizophrenia-risk alleles, which increased the risk for schizophrenia, potentially decreased the *KTN1* mRNA expression in BG (e.g., in BRAINEAC), was just a set of volume-shrinking alleles, which well interpreted how a “recessive” association between schizophrenia-risk *KTN1* alleles and reduced BG GMVs co-existed with a “dominant” association between schizophrenia and expanded BG GMVs.

One limitation of our study is that the SNP-schizophrenia, SNP-mRNA and SNP-ICV/GMV/SA/TH associations were analyzed in separate cohorts, confounded potentially by cohort effect. We noticed that the potential regulatory effects of schizophrenia-risk alleles on *KTN1* mRNA expression in putamen were opposite between BRAINEAC and GTEx, which may be attributed to cohort heterogeneity. Studying these associations in the same cohort in the future would address this limitation.

In summary, this is a study on the role of *KTN1* in schizophrenia. We identified a significant, functional, and robust risk variant block at *KTN1* for schizophrenia. It may regulate the risk for schizophrenia, the *KTN1* mRNA expression in the brain (75%), the GMVs of subcortical structures, and the volumes of around 50% of cortices throughout the brain. It played a “dominant’ role in some specific cortices, but possible “recessive” roles in other cortices and BG in schizophrenia.

### Ethics approval and consent to participate

All subjects signed written informed consents before participating in the study. All study procedures were reviewed and approved by the Human Investigation Committee of Yale University.

## Supplementary Information

Supplementary Information 1.

Supplementary Information 2.

## Data Availability

The datasets used for the analyses described in this manuscript were obtained from dbGaP at http://www.ncbi.nlm.nih.gov/sites/entrez?Db=gap. The dbGaP accession numbers include phs000021.v3.p2 and phs000687.v1.p1.

## References

[1] Breier A (1992). Brain morphology and schizophrenia. A magnetic resonance imaging study of limbic, prefrontal cortex, and caudate structures. Arch. Gen. Psychiat..

[2] Hof PR (2003). Loss and altered spatial distribution of oligodendrocytes in the superior frontal gyrus in schizophrenia. Biol. Psychiat..

[3] Bonilha L (2008). Neurocognitive deficits and prefrontal cortical atrophy in patients with schizophrenia. Schizophr. Res..

[4] Tully LM, Lincoln SH, Liyanage-Don N, Hooker CI (2014). Impaired cognitive control mediates the relationship between cortical thickness of the superior frontal gyrus and role functioning in schizophrenia. Schizophr. Res..

[5] Zhang R, Picchioni M, Allen P, Toulopoulou T (2016). Working memory in unaffected relatives of patients with schizophrenia: A meta-analysis of functional magnetic resonance imaging studies. Schizophr. Bull..

[6] Kikinis Z (2010). Gray matter volume reduction in rostral middle frontal gyrus in patients with chronic schizophrenia. Schizophr. Res..

[7] Guo X (2014). Hippocampal and orbital inferior frontal gray matter volume abnormalities and cognitive deficit in treatment-naive, first-episode patients with schizophrenia. Schizophr. Res..

[8] Riffkin J (2005). A manual and automated MRI study of anterior cingulate and orbito-frontal cortices, and caudate nucleus in obsessive-compulsive disorder: Comparison with healthy controls and patients with schizophrenia. Psychiatry Res..

[9] Iwashiro N (2012). Localized gray matter volume reductions in the pars triangularis of the inferior frontal gyrus in individuals at clinical high-risk for psychosis and first episode for schizophrenia. Schizophr. Res..

[10] Salgado-Pineda P (2014). Structural abnormalities in schizophrenia: further evidence on the key role of the anterior cingulate cortex. Neuropsychobiology.

[11] Onitsuka T (2004). Middle and inferior temporal gyrus gray matter volume abnormalities in chronic schizophrenia: an MRI study. Am. J. Psychiatry.

[12] Kuroki N (2006). Middle and inferior temporal gyrus gray matter volume abnormalities in first-episode schizophrenia: an MRI study. Am. J. Psychiat..

[13] Mennigen E (2019). Positive and general psychopathology associated with specific gray matter reductions in inferior temporal regions in patients with schizophrenia. Schizophr. Res..

[14] Guo W (2014). Decreased gray matter volume in the left middle temporal gyrus as a candidate biomarker for schizophrenia: a study of drug naive, first-episode schizophrenia patients and unaffected siblings. Schizophr. Res..

[15] Cui Y (2018). Auditory verbal hallucinations are related to cortical thinning in the left middle temporal gyrus of patients with schizophrenia. Psychol. Med..

[16] Narayanaswamy JC, Kalmady SV, Venkatasubramanian G, Gangadhar BN (2015). Clinical correlates of superior temporal gyrus volume abnormalities in antipsychotic-naive schizophrenia. J. Neuropsychiatry Clin. Neurosci..

[17] Ohi K (2016). Structural alterations of the superior temporal gyrus in schizophrenia: Detailed subregional differences. Eur. Psychiatry..

[18] Walton E (2017). Positive symptoms associate with cortical thinning in the superior temporal gyrus via the ENIGMA Schizophrenia consortium. Acta Psychiatr. Scand..

[19] Glausier JR, Lewis DA (2013). Dendritic spine pathology in schizophrenia. Neuroscience.

[20] Moyer CE, Shelton MA, Sweet RA (2015). Dendritic spine alterations in schizophrenia. Neurosci. Lett..

[21] Shelton MA (2015). Loss of Microtubule-Associated Protein 2 Immunoreactivity Linked to Dendritic Spine Loss in Schizophrenia. Biol. Psychiat..

[22] Sweet RA, Henteleff RA, Zhang W, Sampson AR, Lewis DA (2009). Reduced dendritic spine density in auditory cortex of subjects with schizophrenia. Neuropsychopharmacology.

[23] Forlim CG (2020). Reduced resting-state connectivity in the precuneus is correlated with apathy in patients with Schizophrenia. Sci. Rep..

[24] Lee CU (2002). Fusiform gyrus volume reduction in first-episode schizophrenia: A magnetic resonance imaging study. Arch. Gen. Psychiatry.

[25] Takahashi T (2006). Temporal lobe gray matter in schizophrenia spectrum: A volumetric MRI study of the fusiform gyrus, parahippocampal gyrus, and middle and inferior temporal gyri. Schizophr. Res..

[26] Takahashi T (2011). A follow-up MRI study of the fusiform gyrus and middle and inferior temporal gyri in schizophrenia spectrum. Prog. Neuropsychopharmacol. Biol. Psychiatry.

[27] Schultz CC (2010). Increased parahippocampal and lingual gyrification in first-episode schizophrenia. Schizophr. Res..

[28] Yu T (2018). Decreased gray matter volume of cuneus and lingual gyrus in schizophrenia patients with tardive dyskinesia is associated with abnormal involuntary movement. Sci. Rep..

[29] Kim JJ (2003). Morphometric abnormality of the insula in schizophrenia: A comparison with obsessive-compulsive disorder and normal control using MRI. Schizophr. Res..

[30] Shepherd AM, Matheson SL, Laurens KR, Carr VJ, Green MJ (2012). Systematic meta-analysis of insula volume in schizophrenia. Biol. Psychiat..

[31] Virupaksha HS (2012). Volume and asymmetry abnormalities of insula in antipsychotic-naive schizophrenia: a 3-tesla magnetic resonance imaging study. Indian J. Psychol. Med..

[32] Onay A, Yapici Eser H, Ulasoglu Yildiz C, Aslan S, Tali ET (2017). A combined VBM and DTI study of schizophrenia: bilateral decreased insula volume and cerebral white matter disintegrity corresponding to subinsular white matter projections unlinked to clinical symptomatology. Diagn. Interv. Radiol..

[33] Caldiroli A (2018). The relationship of IQ and emotional processing with insula volume in schizophrenia. Schizophr. Res..

[34] Bois C (2015). Hippocampal, amygdala and nucleus accumbens volume in first-episode schizophrenia patients and individuals at high familial risk: A cross-sectional comparison. Schizophr. Res..

[35] Rich AM (2016). Amygdala volume is reduced in early course schizophrenia. Psychiat. Res. Neuroimag..

[36] Tesli N (2020). Hippocampal subfield and amygdala nuclei volumes in schizophrenia patients with a history of violence. Eur. Arch. Psychiat. Clin. Neurosci..

[37] Sauras R (2017). Volumetric and morphological characteristics of the hippocampus are associated with progression to schizophrenia in patients with first-episode psychosis. Eur. Psychiat..

[38] Zheng F (2019). Study on the sub-regions volume of hippocampus and amygdala in schizophrenia. Quant Imaging Med. Surg..

[39] Huang X (2017). Decreased left putamen and thalamus volume correlates with delusions in first-episode schizophrenia patients. Front Psychiatry.

[40] Hokama H (1995). Caudate, putamen, and globus pallidus volume in schizophrenia: a quantitative MRI study. Psychiatry Res..

[41] Bridle N (2002). Thalamic and caudate volumes in monozygotic twins discordantfor schizophrenia. Aust. N. Z. J. Psychiatry.

[42] Juuhl-Langseth M (2012). Comprehensive segmentation of subcortical brain volumes in early onset schizophrenia reveals limited structural abnormalities. Psychiatry Res..

[43] Juuhl-Langseth M (2015). Impaired verbal learning is associated with larger caudate volumes in early onset schizophrenia spectrum disorders. PLoS ONE.

[44] Buchsbaum MS (2003). Caudate and putamen volumes in good and poor outcome patients with schizophrenia. Schizophr. Res..

[45] Li M (2012). Volume increases in putamen associated with positive symptom reduction in previously drug-naive schizophrenia after 6 weeks antipsychotic treatment. Psychol. Med..

[46] Spinks R (2005). Globus pallidus volume is related to symptom severity in neuroleptic naive patients with schizophrenia. Schizophr. Res..

[47] Lauer M, Senitz D, Beckmann H (2001). Increased volume of the nucleus accumbens in schizophrenia. J. Neural Transm..

[48] Heckers S, Konradi C (2013). Substantia nigra hyperactivity in schizophrenia. Biol. Psychiat..

[49] Williams MR (2014). Neuropathological changes in the substantia nigra in schizophrenia but not depression. Eur. Arch. Psychiatry Clin. Neurosci..

[50] Mitelman SA (2009). Poor outcome in chronic schizophrenia is associated with progressive loss of volume of the putamen. Schizophr. Res..

[51] Jacobsen LK, Giedd JN, Gottschalk C, Kosten TR, Krystal JH (2001). Quantitative morphology of the caudate and putamen in patients with cocaine dependence. Am. J. Psychiatry.

[52] Harris GJ (1992). Putamen volume reduction on magnetic resonance imaging exceeds caudate changes in mild Huntington's disease. Ann Neurol.

[53] Mahone EM (2016). Anomalous Putamen volume in children with complex motor stereotypies. Pediatr Neurol.

[54] Hibar DP (2015). Common genetic variants influence human subcortical brain structures. Nature.

[55] Kumar J, Yu H, Sheetz MP (1995). Kinectin, an essential anchor for kinesin-driven vesicle motility. Science.

[56] Zhang X (2010). Kinectin-mediated endoplasmic reticulum dynamics supports focal adhesion growth in the cellular lamella. J. Cell Sci..

[57] Toyoshima I, Sheetz MP (1996). Kinectin distribution in chicken nervous system. Neurosci. Lett..

[58] Xu B (2017). Impact of a common genetic variation associated with Putamen volume on neural mechanisms of attention-deficit/hyperactivity disorder. J. Am. Acad. Child. Adolesc. Psychiatry.

[59] Luo X (2020). KTN1 variants and risk for attention deficit hyperactivity disorder. Am. J. Med. Genet. B Neuropsychiatr. Genet..

[60] Nalls MA (2014). Large-scale meta-analysis of genome-wide association data identifies six new risk loci for Parkinson's disease. Nat. Genet..

[61] Chang D (2017). A meta-analysis of genome-wide association studies identifies 17 new Parkinson's disease risk loci. Nat. Genet..

[62] van Dijk KD (2012). The proteome of the locus ceruleus in Parkinson's disease: relevance to pathogenesis. Brain Pathol..

[63] Mao Q (2020). KTN1 variants underlying Putamen gray matter volumes and Parkinson's disease. Front. Neurosci..

[64] Li Y (2016). A population-based study of four genes associated with heroin addiction in Han Chinese. PLoS ONE.

[65] Stringer S (2016). Genome-wide association study of lifetime cannabis use based on a large meta-analytic sample of 32 330 subjects from the International Cannabis Consortium. Transl. Psychiatry.

[66] Luo, X. *et al.* Significant, replicable, and functional associations between KTN1 variants and alcohol and drug codependence. *Addict Biol*, e12888 (2020). 10.1111/adb.12888 PMC7641293 32115811

[67] Han L (2017). Potential contribution of the neurodegenerative disorders risk loci to cognitive performance in an elderly male gout population. Medicine.

[68] American Psychiatric Association. *Diagnostic and statistical manual of mental disorders*. fourth edition edn, (American Psychiatric Press, 1994).

[69] Gain Collaborative Research Group *et al.* New models of collaboration in genome-wide association studies: The Genetic Association Information Network. *Nat. Genet.* **39**, 1045–1051 (2007). 10.1038/ng2127 17728769

[70] O'Donovan MC (2008). Identification of loci associated with schizophrenia by genome-wide association and follow-up. Nat. Genet..

[71] International Schizophrenia Consortium *et al.* Common polygenic variation contributes to risk of schizophrenia and bipolar disorder. *Nature* **460**, 748–752 (2009). 10.1038/nature08185 PMC3912837 19571811

[72] Stefansson H (2009). Common variants conferring risk of schizophrenia. Nature.

[73] Fromer M (2014). De novo mutations in schizophrenia implicate synaptic networks. Nature.

[74] Wang Z (2022). An independent, replicable, functional and significant risk variant block at intron 3 of CACNA1C for schizophrenia. Aust N Z J Psychiatry.

[75] Howie BN, Donnelly P, Marchini J (2009). A flexible and accurate genotype imputation method for the next generation of genome-wide association studies. PLoS Genet.

[76] Zuo L (2012). Genome-wide association study of alcohol dependence implicates KIAA0040 on chromosome 1q. Neuropsychopharmacology.

[77] Purcell S (2007). PLINK: a tool set for whole-genome association and population-based linkage analyses. Am. J. Hum. Genet..

[78] Lam M (2019). Comparative genetic architectures of schizophrenia in East Asian and European populations. Nat. Genet..

[79] Ramasamy A (2014). Genetic variability in the regulation of gene expression in ten regions of the human brain. Nat. Neurosci..

[80] GTEx Consortium. The Genotype-Tissue Expression (GTEx) project. *Nat. Genet.* **45**, 580–585 (2013). 10.1038/ng.2653 PMC4010069 23715323

[81] Adams HH (2016). Novel genetic loci underlying human intracranial volume identified through genome-wide association. Nat. Neurosci..

[82] Satizabal CL (2019). Genetic architecture of subcortical brain structures in 38,851 individuals. Nat. Genet..

[83] Patenaude B, Smith SM, Kennedy DN, Jenkinson M (2011). A Bayesian model of shape and appearance for subcortical brain segmentation. Neuroimage.

[84] Fischl B (2012). FreeSurfer. NeuroImage.

[85] Grasby KL (2020). The genetic architecture of the human cerebral cortex. Science.

[86] Desikan RS (2006). An automated labeling system for subdividing the human cerebral cortex on MRI scans into gyral based regions of interest. Neuroimage.

[87] Rakic P (1988). Specification of cerebral cortical areas. Science.

[88] Nakai T, Kato C, Matsuo K (2005). An FMRI study to investigate auditory attention: a model of the cocktail party phenomenon. Magn. Reson. Med. Sci..

[89] Babiloni C (2005). Human cortical responses during one-bit delayed-response tasks: an fMRI study. Brain Res. Bull..

[90] Fincham JM, Carter CS, van Veen V, Stenger VA, Anderson JR (2002). Neural mechanisms of planning: a computational analysis using event-related fMRI. Proc. Natl. Acad. Sci. USA.

[91] Bahlmann J, Aarts E, D'Esposito M (2015). Influence of motivation on control hierarchy in the human frontal cortex. J. Neurosci..

[92] Stuss DT, Gow CA, Hetherington CR (1992). "No longer Gage": frontal lobe dysfunction and emotional changes. J. Consult Clin. Psychol..

[93] Kerestes R (2012). Abnormal prefrontal activity subserving attentional control of emotion in remitted depressed patients during a working memory task with emotional distracters. Psychol. Med..

[94] Goldberg II, Harel M, Malach R (2006). When the brain loses its self: prefrontal inactivation during sensorimotor processing. Neuron.

[95] Stocco A, Lebiere C, Anderson JR (2010). Conditional routing of information to the cortex: A model of the basal ganglia's role in cognitive coordination. Psychol Rev.

[96] Frank MJ, O'Reilly RC (2006). A mechanistic account of striatal dopamine function in human cognition: psychopharmacological studies with cabergoline and haloperidol. Behav Neurosci.

[97] Ikemoto S, Yang C, Tan A (2015). Basal ganglia circuit loops, dopamine and motivation: A review and enquiry. Behav. Brain Res..

[98] Inta D, Meyer-Lindenberg A, Gass P (2011). Alterations in postnatal neurogenesis and dopamine dysregulation in schizophrenia: a hypothesis. Schizophr. Bull..

[99] Jou RJ, Minshew NJ, Keshavan MS, Vitale MP, Hardan AY (2010). Enlarged right superior temporal gyrus in children and adolescents with autism. Brain Res..

[100] Bigler ED (2007). Superior temporal gyrus, language function, and autism. Dev. Neuropsychol..

[101] Kjaer TW, Nowak M, Lou HC (2002). Reflective self-awareness and conscious states: PET evidence for a common midline parietofrontal core. Neuroimage.

[102] Fletcher PC (1995). The mind's eye–precuneus activation in memory-related imagery. Neuroimage.

[103] Oshio R (2010). Differential effect of double-pulse TMS applied to dorsal premotor cortex and precuneus during internal operation of visuospatial information. Neuroimage.

[104] Jalbrzikowski M (2013). Structural abnormalities in cortical volume, thickness, and surface area in 22q112 microdeletion syndrome: Relationship with psychotic symptoms. NeuroImage. Clin..

