# Supplementary figures and images for: A significant, functional and replicable risk KTN1 variant block for schizophrenia
Source: Sci Rep. 2023 Mar 8;13:3890. doi: 10.1038/s41598-023-27448-z (PMC9995530; doi:10.1038/s41598-023-27448-z)

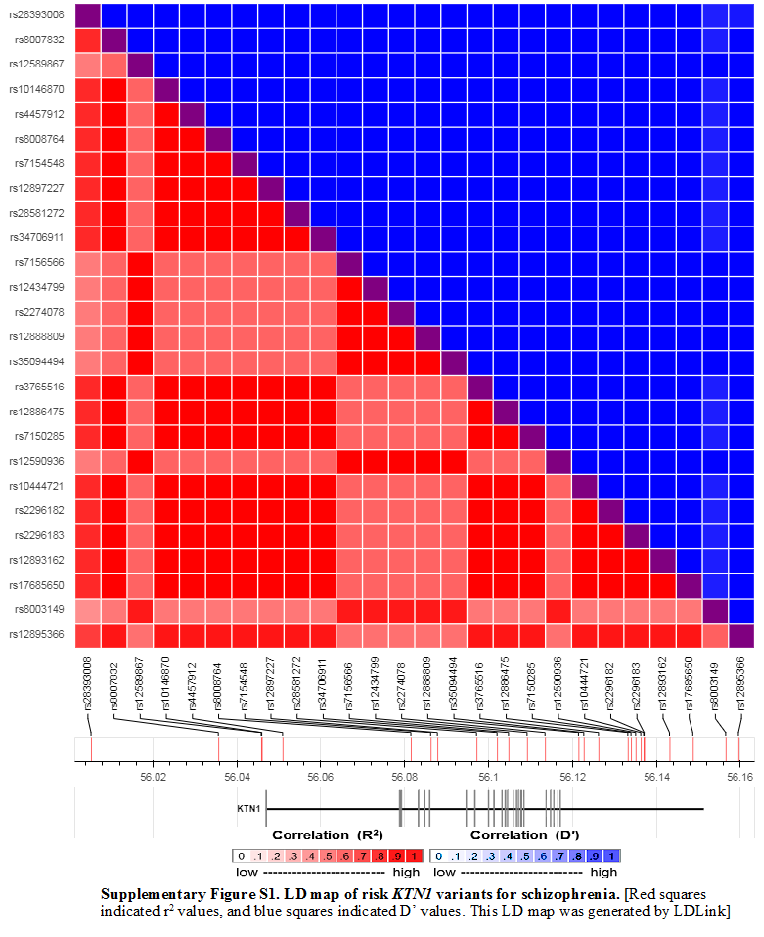

Supplement: Supplementary file 1 — Supplementary Information 1. [file 41598_2023_27448_MOESM1_ESM.png]
