# Supplementary material for: A significant, functional and replicable risk KTN1 variant block for schizophrenia
Source: Sci Rep. 2023 Mar 8;13:3890. doi: 10.1038/s41598-023-27448-z (PMC9995530; doi:10.1038/s41598-023-27448-z)
Supplement: Supplementary file 2 — Supplementary Information 2. [file 41598_2023_27448_MOESM2_ESM.docx]

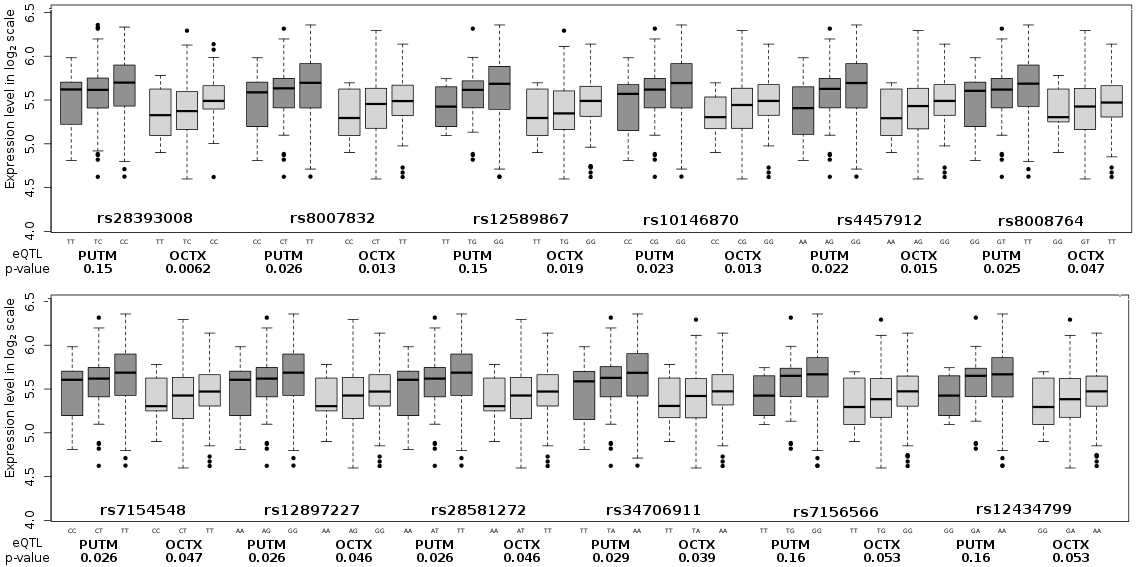

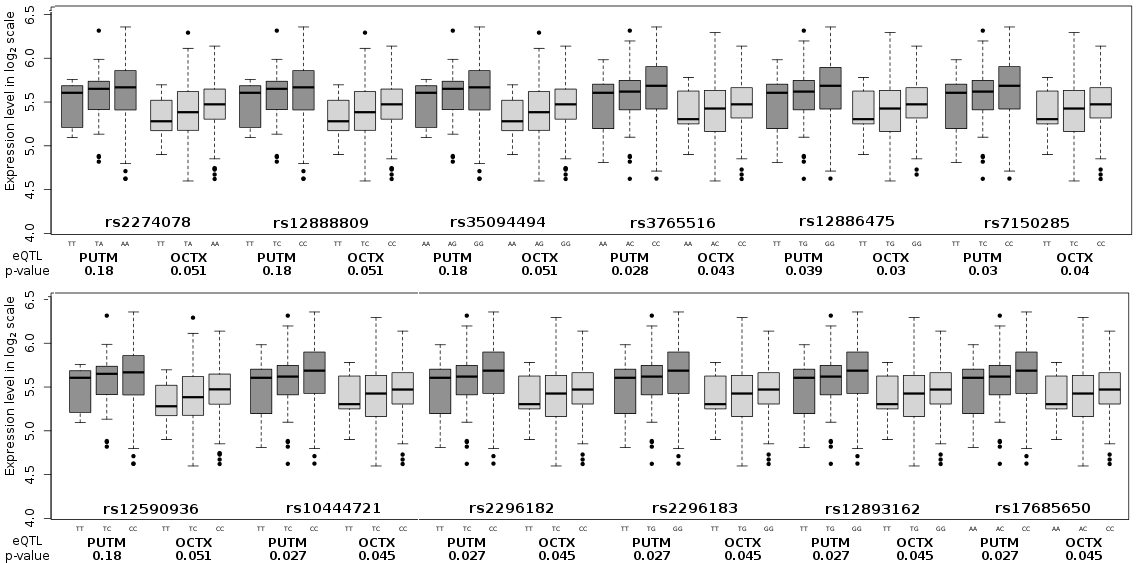

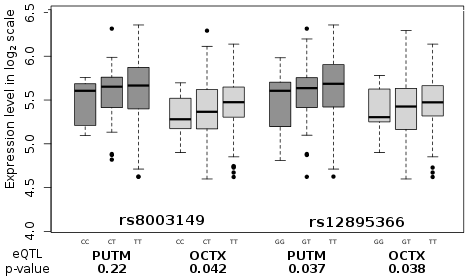


**Supplementary Figure S2. SNP-mRNA associations in BRAINEAC** [P-values for these associations refer to Table 2. X-axis, mRNA expression level; Y-axis, brain regions]

|  |  | Brain regions | | | | | | | | | | | |
| --- | --- | --- | --- | --- | --- | --- | --- | --- | --- | --- | --- | --- | --- |
| SNP | Effective allele | 1 | 2 | 3 | 4 | 5 | 6 | 7 | 8 | 9 | 10 | 11 | 12 |
| rs28393008 | T |  |  |  |  |  |  |  |  |  |  |  |  |
| rs8007832 | C |  |  |  |  |  |  |  |  |  |  |  |  |
| rs12589867 | T |  |  |  |  |  |  |  |  |  |  |  |  |
| rs10146870 | C |  |  |  |  |  |  |  |  |  |  |  |  |
| rs4457912 | A |  |  |  |  |  |  |  |  |  |  |  |  |
| rs8008764 | G |  |  |  |  |  |  |  |  |  |  |  |  |
| rs7154548 | C |  |  |  |  |  |  |  |  |  |  |  |  |
| rs12897227 | A |  |  |  |  |  |  |  |  |  |  |  |  |
| rs28581272 | A |  |  |  |  |  |  |  |  |  |  |  |  |
| rs34706911 | T |  |  |  |  |  |  |  |  |  |  |  |  |
| rs7156566 | T |  |  |  |  |  |  |  |  |  |  |  |  |
| rs12434799 | G |  |  |  |  |  |  |  |  |  |  |  |  |
| rs2274078 | T |  |  |  |  |  |  |  |  |  |  |  |  |
| rs12888809 | T |  |  |  |  |  |  |  |  |  |  |  |  |
| rs35094494 | A |  |  |  |  |  |  |  |  |  |  |  |  |
| rs3765516 | A |  |  |  |  |  |  |  |  |  |  |  |  |
| rs12886475 | T |  |  |  |  |  |  |  |  |  |  |  |  |
| rs7150285 | T |  |  |  |  |  |  |  |  |  |  |  |  |
| rs12590936 | T |  |  |  |  |  |  |  |  |  |  |  |  |
| rs10444721 | T |  |  |  |  |  |  |  |  |  |  |  |  |
| rs2296182 | T |  |  |  |  |  |  |  |  |  |  |  |  |
| rs2296183 | T |  |  |  |  |  |  |  |  |  |  |  |  |
| rs12893162 | A |  |  |  |  |  |  |  |  |  |  |  |  |
| rs17685650 | A |  |  |  |  |  |  |  |  |  |  |  |  |
| rs8003149 | C |  |  |  |  |  |  |  |  |  |  |  |  |
| rs12895366 | G |  |  |  |  |  |  |  |  |  |  |  |  |

**Supplementary Figure S3. SNP-mRNA associations in GTEx** [P-values for these associations refer to Table 2. The color blue-yellow-red corresponds to the low-median-high normalized effect size (NES) value (all NES>0). Brain regions 1-12: Cortex, Frontal cortex, Anterior cingulated, Amygdala, Hippocampus, Hypothalamus, Caudate, Putamen, Nucleus accumbens, Substantia nigra, Cerebellar Hemisphere, and Cerebellum]

| SNP | Effective allele | ICV |
| --- | --- | --- |
| rs28393008 | T |  |
| rs8007832 | C |  |
| rs12589867 | T |  |
| rs10146870 | C |  |
| rs4457912 | A |  |
| rs8008764 | G |  |
| rs7154548 | C |  |
| rs12897227 | A |  |
| rs28581272 | A |  |
| rs34706911 | T |  |
| rs7156566 | T |  |
| rs12434799 | G |  |
| rs2274078 | T |  |
| rs12888809 | T |  |
| rs35094494 | A |  |
| rs3765516 | A |  |
| rs12886475 | T |  |
| rs7150285 | T |  |
| rs12590936 | T |  |
| rs10444721 | T |  |
| rs2296182 | T |  |
| rs2296183 | T |  |
| rs12893162 | A |  |
| rs17685650 | A |  |
| rs8003149 | C |  |
| rs12895366 | G |  |

**Supplementary Figure S4. SNP-ICV (intracranial volume) associations** [P-values for these associations refer to Table 3. The color blue-yellow-red corresponds to the low-median-high Z-score (all Z>0)]

|  |  | Brain regions | | | | | | | |
| --- | --- | --- | --- | --- | --- | --- | --- | --- | --- |
|  |  | Putamen | | Caudate | | Pallidum | | Accumbens | |
| SNP | Effective allele | #1 | #2 | #1 | #2 | #1 | #2 | #1 | #2 |
| rs28393008 | C |  |  |  |  |  |  |  |  |
| rs8007832 | T |  |  |  |  |  |  |  |  |
| rs12589867 | G |  |  |  |  |  |  |  |  |
| rs10146870 | G |  |  |  |  |  |  |  |  |
| rs4457912 | G |  |  |  |  |  |  |  |  |
| rs8008764 | T |  |  |  |  |  |  |  |  |
| rs7154548 | T |  |  |  |  |  |  |  |  |
| rs12897227 | G |  |  |  |  |  |  |  |  |
| rs28581272 | T |  |  |  |  |  |  |  |  |
| rs34706911 | A |  |  |  |  |  |  |  |  |
| rs7156566 | G |  |  |  |  |  |  |  |  |
| rs12434799 | A |  |  |  |  |  |  |  |  |
| rs2274078 | A |  |  |  |  |  |  |  |  |
| rs12888809 | C |  |  |  |  |  |  |  |  |
| rs35094494 | G |  |  |  |  |  |  |  |  |
| rs3765516 | C |  |  |  |  |  |  |  |  |
| rs12886475 | G |  |  |  |  |  |  |  |  |
| rs7150285 | C |  |  |  |  |  |  |  |  |
| rs12590936 | C |  |  |  |  |  |  |  |  |
| rs10444721 | C |  |  |  |  |  |  |  |  |
| rs2296182 | C |  |  |  |  |  |  |  |  |
| rs2296183 | G |  |  |  |  |  |  |  |  |
| rs12893162 | G |  |  |  |  |  |  |  |  |
| rs17685650 | C |  |  |  |  |  |  |  |  |
| rs8003149 | T |  |  |  |  |  |  |  |  |
| rs12895366 | T |  |  |  |  |  |  |  |  |

**Supplementary Figure S5. SNP-GMV associations** [P-values and Cohorts #1 and #2 for these associations refer to Table 3. The color blue-yellow-red corresponds to the low-median-high Z-score (Z>0 in Cohort #1) or β value (β>0 in Cohort #2); white color indicates missing values]

|  |  | Regions | | |  | Regions | | |
| --- | --- | --- | --- | --- | --- | --- | --- | --- |
| SNP | Effective allele | 1 | 2 | 3 | Effective allele | 4 | 5 | 6 |
| rs28393008 | T |  |  |  | C |  |  |  |
| rs8007832 | C |  |  |  | T |  |  |  |
| rs12589867 | T |  |  |  | G |  |  |  |
| rs10146870 | C |  |  |  | G |  |  |  |
| rs4457912 | A |  |  |  | G |  |  |  |
| rs8008764 | G |  |  |  | T |  |  |  |
| rs7154548 | C |  |  |  | T |  |  |  |
| rs12897227 | A |  |  |  | G |  |  |  |
| rs28581272 | A |  |  |  | T |  |  |  |
| rs34706911 | T |  |  |  | A |  |  |  |
| rs7156566 | T |  |  |  | G |  |  |  |
| rs12434799 | G |  |  |  | A |  |  |  |
| rs2274078 | T |  |  |  | A |  |  |  |
| rs12888809 | T |  |  |  | C |  |  |  |
| rs35094494 | A |  |  |  | G |  |  |  |
| rs3765516 | A |  |  |  | C |  |  |  |
| rs12886475 | T |  |  |  | G |  |  |  |
| rs12590936 | T |  |  |  | C |  |  |  |
| rs10444721 | T |  |  |  | C |  |  |  |
| rs2296182 | T |  |  |  | C |  |  |  |
| rs2296183 | T |  |  |  | G |  |  |  |
| rs12893162 | A |  |  |  | G |  |  |  |
| rs17685650 | A |  |  |  | C |  |  |  |
| rs8003149 | C |  |  |  | T |  |  |  |
| rs12895366 | G |  |  |  | T |  |  |  |

**Supplementary Figure S6. SNP-SA (cortical surface area) associations** [P-values for these associations refer to Table 4. The color blue-yellow-red corresponds to the low-median-high β value (all β>0). Brain regions 1-6: Whole, Lingual, Middle temporal, Precuneus, Insula and Frontal pole]

.

|  |  | Brain regions | | | | | | | |  |  |
| --- | --- | --- | --- | --- | --- | --- | --- | --- | --- | --- | --- |
| SNP | Effective allele | 1 | 2 | 3 | 4 | 5 | 6 | 7 | 8 | Effective allele | 9 |
| rs28393008 | T |  |  |  |  |  |  |  |  | C |  |
| rs8007832 | C |  |  |  |  |  |  |  |  | T |  |
| rs12589867 | T |  |  |  |  |  |  |  |  | G |  |
| rs10146870 | C |  |  |  |  |  |  |  |  | G |  |
| rs4457912 | A |  |  |  |  |  |  |  |  | G |  |
| rs8008764 | G |  |  |  |  |  |  |  |  | T |  |
| rs7154548 | C |  |  |  |  |  |  |  |  | T |  |
| rs12897227 | A |  |  |  |  |  |  |  |  | G |  |
| rs28581272 | A |  |  |  |  |  |  |  |  | T |  |
| rs34706911 | T |  |  |  |  |  |  |  |  | A |  |
| rs7156566 | T |  |  |  |  |  |  |  |  | G |  |
| rs12434799 | G |  |  |  |  |  |  |  |  | A |  |
| rs2274078 | T |  |  |  |  |  |  |  |  | A |  |
| rs12888809 | T |  |  |  |  |  |  |  |  | C |  |
| rs35094494 | A |  |  |  |  |  |  |  |  | G |  |
| rs3765516 | A |  |  |  |  |  |  |  |  | C |  |
| rs12886475 | T |  |  |  |  |  |  |  |  | G |  |
| rs12590936 | T |  |  |  |  |  |  |  |  | C |  |
| rs10444721 | T |  |  |  |  |  |  |  |  | C |  |
| rs2296182 | T |  |  |  |  |  |  |  |  | C |  |
| rs2296183 | T |  |  |  |  |  |  |  |  | G |  |
| rs12893162 | A |  |  |  |  |  |  |  |  | G |  |
| rs17685650 | A |  |  |  |  |  |  |  |  | C |  |
| rs8003149 | C |  |  |  |  |  |  |  |  | T |  |
| rs12895366 | G |  |  |  |  |  |  |  |  | T |  |

**Supplementary Figure S7. SNP-TH (cortical thickness) associations** [P-values for these associations refer to Table 4. The color blue-yellow-red corresponds to the low-median-high β value (all β>0). Brain regions 1-9: Frontal pole, Frontal pole, Superior frontal, Rostral middle frontal, Lateral orbito-frontal, Pars opercularis, Isthmus cingulate, Inferior temporal, and Pericalcarine]

|  |  |  |  |  |  |  |  |  |
| --- | --- | --- | --- | --- | --- | --- | --- | --- |
